# Are there distinct levels of language comprehension in autistic individuals – cluster analysis

**DOI:** 10.1038/s44184-024-00062-1

**Published:** 2024-04-10

**Authors:** Andrey Vyshedskiy, Rohan Venkatesh, Edward Khokhlovich

**Affiliations:** 1 Boston University, Boston, MA 02215 USA; 2 Independent researcher, Newton, MA 02459 USA

**Keywords:** Human behaviour, Autism spectrum disorders

## Abstract

Autism is a neurodevelopmental condition characterized by deficits in social communication. We assessed 14-language comprehension abilities in 31,845 autistic individuals 4 to 21 years of age using parent-generated reports. Data-driven cluster analysis identified three distinct levels of language comprehension: (1) individuals in the command-language-phenotype were limited to comprehension of simple commands; (2) individuals in the modifier-language-phenotype showed additional comprehension of color, size, and number modifiers; 3) individuals in the most-advanced syntactic-language-phenotype added comprehension of spatial prepositions, verb tenses, flexible syntax, possessive pronouns, and complex narratives. The observation of three distinct language levels was consistent across different age groups. Autistic individuals’ communication level is currently commonly characterized as *nonverbal*, *minimally-verbal*, or *verbal*. This one-dimensional description is not ideal for characterizing an individual’s communication ability. In fact, a *nonverbal* individual with syntactic-language-phenotype may have normal ability to communicate albeit nonverbally, while a *verbal* person with command-language-phenotype does not have a normal ability to communicate by any means. Identification of the three distinct language-comprehension-phenotypes provides an opportunity to enhance characterization of individuals’ communication level. A composite description in terms of both, verbal abilities and a language-comprehension-level, will not only be more precise, but can improve language therapy by focusing it on both aspects of language development.

## Introduction

Autism Spectrum Disorder (ASD) is a neurodevelopmental condition marked by impairments in social communication and restricted, repetitive patterns of behavior^1^. Evidence from multiple studies suggests that language deficits may be a core component of autism^2–4^. Moreover, language comprehension is usually more impaired than expressive language^5–11^. Despite these observations, communication level in individuals with ASD is commonly (albeit more informally, than formerly) characterized in terms of their verbal level (*nonverbal*, *minimally verbal*, or *verbal*), and their language comprehension ability is left unclassified. If there was a clear and objective way to classify an individual’s language comprehension level, such a classification system could improve description of individuals’ communication skills, facilitate their language therapy, and improve outcomes.

Over a decade ago a parent-reported language comprehension survey Mental Synthesis Evaluation Checklist (MSEC) was developed by our group with a specific goal: to target higher aspects of language^3,12^. MSEC uses the following questions to assess language comprehension: (1) [My child] Understands simple stories that are read aloud; (2) Understands elaborate fairy tales that are read aloud (i.e., stories describing FANTASY creatures); (3) Understands some simple modifiers (i.e., green apple vs. red apple or big apple vs. small apple); (4) Understands several modifiers in a sentence (i.e., small green apple); 5) Understands size (can select the largest/smallest object out of a collection of objects); (6) Understands possessive pronouns (i.e. your apple vs. her apple); (7) Understands spatial prepositions (i.e., put the apple ON TOP of the box vs. INSIDE the box vs. BEHIND the box); (8) Understands verb tenses (i.e., I will eat an apple vs. I ate an apple); (9) Understands the change in meaning when the order of words is changed (i.e., understands the difference between ‘a cat ate a mouse’ vs. ‘a mouse ate a cat’); 10) Understands explanations about people, objects or situations beyond the immediate surroundings (e.g., “Mom is walking the dog,” “The snow has turned to water”).

The MSEC survey was administered along with several other surveys within an app, which is popular among individuals with ASD (approximately 65% of app users are diagnosed with ASD^3^). Over 100,000 parents responded to the survey between 2015 and 2022. The language comprehension survey MSEC proved to be an essential outcome measure in multiple studies investigating developmental trajectories in autistic children: (1) MSEC score in autistic children (*N* = 29,138) was significantly different from typically developing children as early as 2 years of age with the ASD diagnosis sensitivity and specificity of 0.71 and 0.88 respectively^3^. The sensitivity and specificity of MSEC improved with age to 0.92/0.95 at 3 years of age; 0.91/1.0 at 4 years of age; 0.93/0.97 at 5 years of age; 0.93/1.0 at 6 years of age; and 0.95/1.0 at 7 years of age. (2) In another 3-year epidemiological study investigating the effect of passive video and television watching (*N* = 3227), shorter video and television watching was associated with 1.4-fold (*p* = 0.0128) greater improvement in the language comprehension score measured by MSEC, but 1.3-fold (*p* = 0.0719) reduction in the expressive language score^13^. Notably, if the MSEC data were not available, the study conclusion would have been that longer television watching time had merely a positive effect on children with ASD, as exhibited by the expressive language subscale. Only with an addition of the language comprehension MSEC scale was it possible to detect the significant negative effect of passive video and television watching. (3) In another 3-year study (*N* = 6454), children who engaged with a therapeutic language intervention increased their language comprehension MSEC score 2.2-fold when compared to children with similar initial evaluations (*p* < 0.0001). At the same time, the difference between the groups in the expressive language score was only 1.4-fold (*p* = 0.0144)^14^. (4) In a 3-year study of the effect of pretend play (*N* = 7069), pretend play was associated with 1.9-fold faster improvement of language comprehension measured by MSEC (*p* < 0.0001), but only 1.4-fold faster improvement of expressive language (*p* < 0.0001)^15^. (5) In a 3-year study of the effect of joint-engagement (*N* = 12,081), longer duration of joint-engagement was associated with 1.4-times faster development of language comprehension measured by MSEC score (*p* = 0.0019). (6) In a 3-year study of the effect of seizures (*N* = 8461), children with no seizures improved their language comprehension measured by MSEC 1.5-times faster than those with seizures (*p* < 0.0001). (7) In a 3-year study of diet and food consumption (*N* = 5553), gluten-free diet was associated with 1.5-fold faster improvement of language comprehension measured by MSEC (*p* < 0.0001), but no significant improvement of expressive language (*p* = 0.5918)^16^. (8) In the same study, meat- and eggs-eating was associated with 1.6-fold faster improvement of language comprehension measured by MSEC (*p* < 0.0001), but only in 1.1-times faster improvement of expressive language (*p* = 0. 0279)^16^. (9) Also in the same study, vegetable-eating was associated with 1.5-fold faster improvement of language comprehension measured by MSEC (*p* < 0.0001), but only in 1.2-times faster improvement of expressive language (*p* = 0. 0137)^16^. The results of these studies demonstrate that language comprehension survey MSEC is well received by parents and provides valuable information on autistic children development.

This study investigates which of the language comprehension abilities manifest concurrently, using unsupervised hierarchical clustering and principal component analysis – statistical techniques highly popular in computational genetics. When several genes are co-expressed, they are usually under the regulatory control of a shared transcription factor^17^. Similar to analysis of co-expressed genes, linguistic abilities that manifest concurrently are likely to have a related regulatory mechanism. Cluster analysis methods automatically organize items based on their similarities, forming tree-like diagrams (called dendrograms). These dendrograms depict the hierarchical connections between groups of items. If any two items/abilities were mediated by the same mechanism, a disruption in that mechanism would result in the absence of both abilities, leading to their clustering within the same group. Conversely, clustering into different groups would suggest separate underlying mechanisms.

Additionally, we conducted a cluster analysis of participants based on their language comprehension phenotype. Previous studies have used cluster analysis to clarify diagnostic heterogeneity in ASD on the basis of core symptoms via different approaches including unsupervised machine learning^18–21^, but never on the basis of language comprehension. It was hypothesized that individuals with ASD can be assigned to subgroups (phenotypes) based on their language comprehension abilities. It is worth noting that in the past, language phenotypes were identified primarily on the bases of expressive language, but not based on language comprehension abilities^22,23^. Existence of such phenotypes would enable better classification of autistic individuals’ communication level, as well as facilitate targeting language comprehension as a standard part of therapeutic intervention.

## Results

### Clusters of language comprehension abilities

**Fig. 1:**
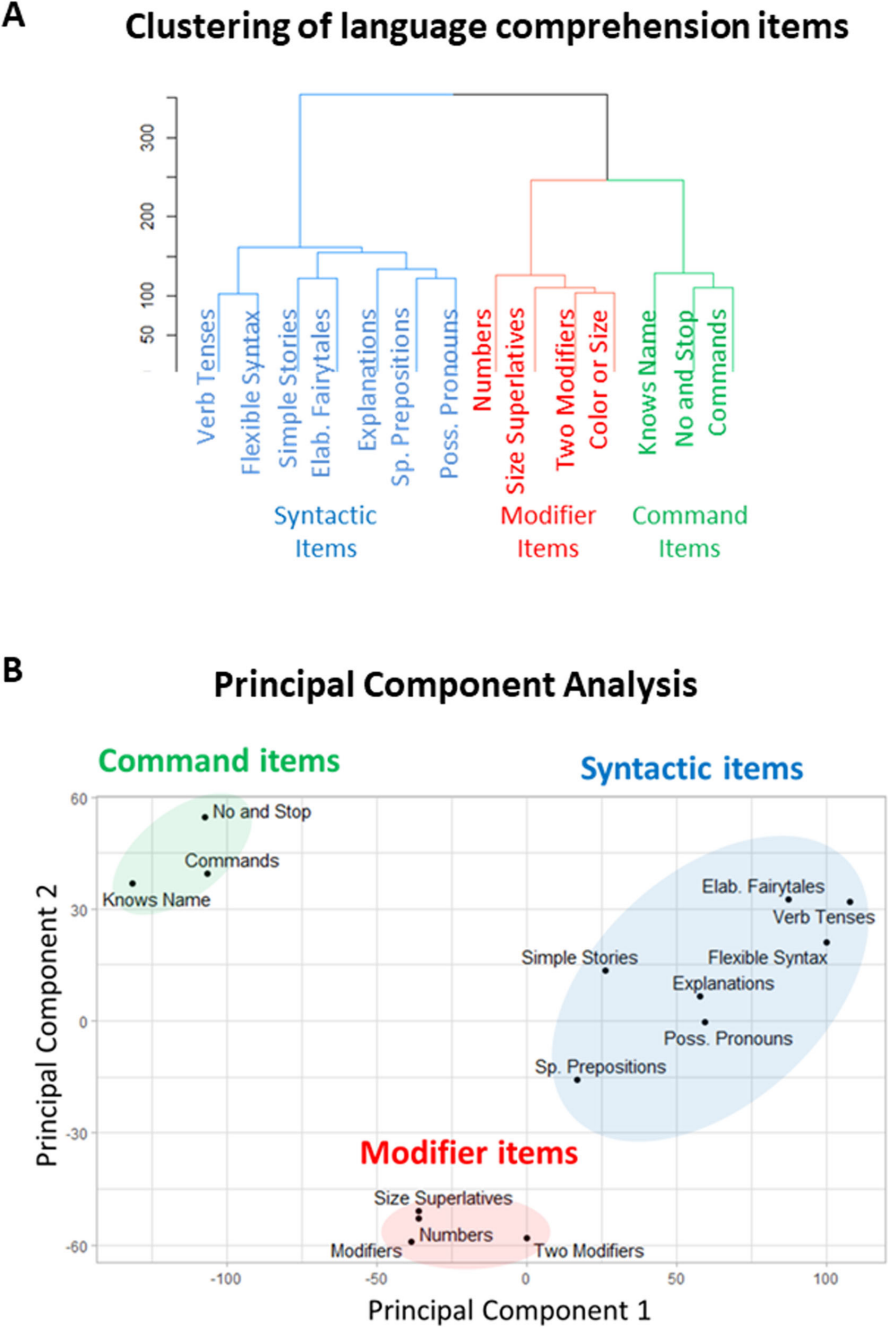
Clustering analysis of language comprehension items. **A** The dendrogram representing the unsupervised hierarchical clustering of language comprehension abilities. **B** Principal component analysis of the 14 language comprehension abilities shows clear separation between command, modifier, and syntactic items. Principal component 1 accounts for 44.9% of the variance in the data. Principal component 2 accounts for 11.7% of the variance in the data.

Caregivers assessed 14 language comprehension abilities (Table 1) in 31,845 autistic individuals. To investigate which abilities were acquired concurrently, we performed unsupervised hierarchical clustering. Unsupervised hierarchical cluster analysis is a data-driven approach that automatically arranges items according to their similarities, forming tree-like diagrams called dendrograms. These dendrograms visually represent the hierarchical relationships among clusters of items. The dendrogram calculated as a result of the unsupervised hierarchical cluster analysis of 14 language comprehension abilities is shown in Fig. 1A. The distance between clusters is determined by the height of the dendrogram branches. The greater the distance, the more dissimilar are the clusters. Three clusters have inter-cluster distances that are considerably greater than distances between subclusters. The first cluster, termed “command language,” includes knowing the name, responding to ‘No’ or ‘Stop’, and following some commands (items 1 to 3 in Table 1). The second cluster, termed “modifier language,” includes understanding color and size modifiers, several modifiers in a sentence, size superlatives, and numbers (items 4 to 7 in Table 1). The third cluster, termed the “syntactic language,” includes understanding of spatial prepositions, verb tenses, flexible syntax, possessive pronouns, explanations about people and situations, simple stories, and elaborate fairytales (items 8 to 14 in Table 1).

**Table 1:** Language comprehension items as they were posed to parents verbatim

	Language comprehension items (verbatim)	Abbreviations used in dendrograms
1	Knows own name	Knows Name
2	Responds to ‘No’ or ‘Stop’	No and Stop
3	Can follow some commands	Commands
4	Understands some simple modifiers (i.e., green apple vs. red apple or big apple vs. small apple)	Color or Size / Modifiers
5	Understands several modifiers in a sentence (i.e., small green apple)	Two Modifiers
6	Understands size (can select the largest/smallest object out of a collection of objects)	Size Superlatives
7	Understands NUMBERS (i.e., two apples vs. three apples)	Numbers
8	Understands spatial prepositions (i.e., put the apple ON TOP of the box vs. INSIDE the box vs. BEHIND the box)	Sp. Prepositions
9	Understands verb tenses (i.e., I will eat an apple vs. I ate an apple)	Verb Tenses
10	Understands simple stories that are read aloud	Simple Stories
11	Understands elaborate fairytales that are read aloud (i.e., stories describing FANTASY creatures)	Elab. Fairytales
12	Understands possessive pronouns (i.e., your apple vs. her apple)	Poss. Pronouns
13	Understands the change in meaning when the order of words is changed (i.e., understands the difference between ‘a cat ate a mouse’ vs. ‘a mouse ate a cat’)	Flexible Syntax
14	Understands explanations about people, objects or situations beyond the immediate surroundings (e.g., “Mom is walking the dog,” “The snow has turned to water”).	Explanations

The answers choices were: very true (0 points), somewhat true (1 point), and not true (2 points). A lower score indicates better language comprehension ability.

The next tenable solution based on the height of dendrogram branches would include five clusters (Fig. 1A: 1) Command items; (2) Modifier items; (3) Explanations, Spatial Prepositions, and Possessive Pronouns; (4) Simple Stories and Elaborate Fairytales; and (5) Verb Tenses and Flexible Syntax). The three-cluster solution is superior to the five-cluster solution because it has greater average inter-cluster distance. We also considered a two-cluster solution (Fig. 1A: 1) Command and Modifier items; (2) Syntactic items). However, some clustering methods (e.g., the “average” clustering methods, Supplementary Fig. 1C) combine the Modifier branch with the Syntactic branch, making the two-cluster solution unstable. Consequently, the three-cluster solution is the most reasonable classification in terms of the average inter-cluster distance and stability.

Reassuringly, the principal component analysis also shows clear separation between Command, Modifier and Syntactic clusters (Fig. 1B). Furthermore, correlation analysis between the 14 language comprehension abilities shows stronger correlation between items within each of the three clusters and weaker correlation between items outside each cluster (Supplementary Fig. 2). Thus, multiple analysis methods corroborate the three clusters solution.

The three-cluster solution was stable across multiple seeds, as well as consistent across different evaluation methods (Ward.D2 method, Supplementary Fig. 1A; Ward.D method, Supplementary Fig. 1B; Average method, Supplementary Fig. 1C; Complete method, Supplementary Fig. 1D; Mcquitty method, Supplementary Fig. 1E), across different age groups (4 to 6 years of age, Supplementary Fig. 3; 6 to 12 years of age, Supplementary Fig. 4; 12 to 21 years of age, Supplementary Fig. 5), and across different time points (first evaluation, Supplementary Fig. 6; last evaluation, Fig. 1).

### Language comprehension phenotypes in individuals with autism

**Fig. 2:**
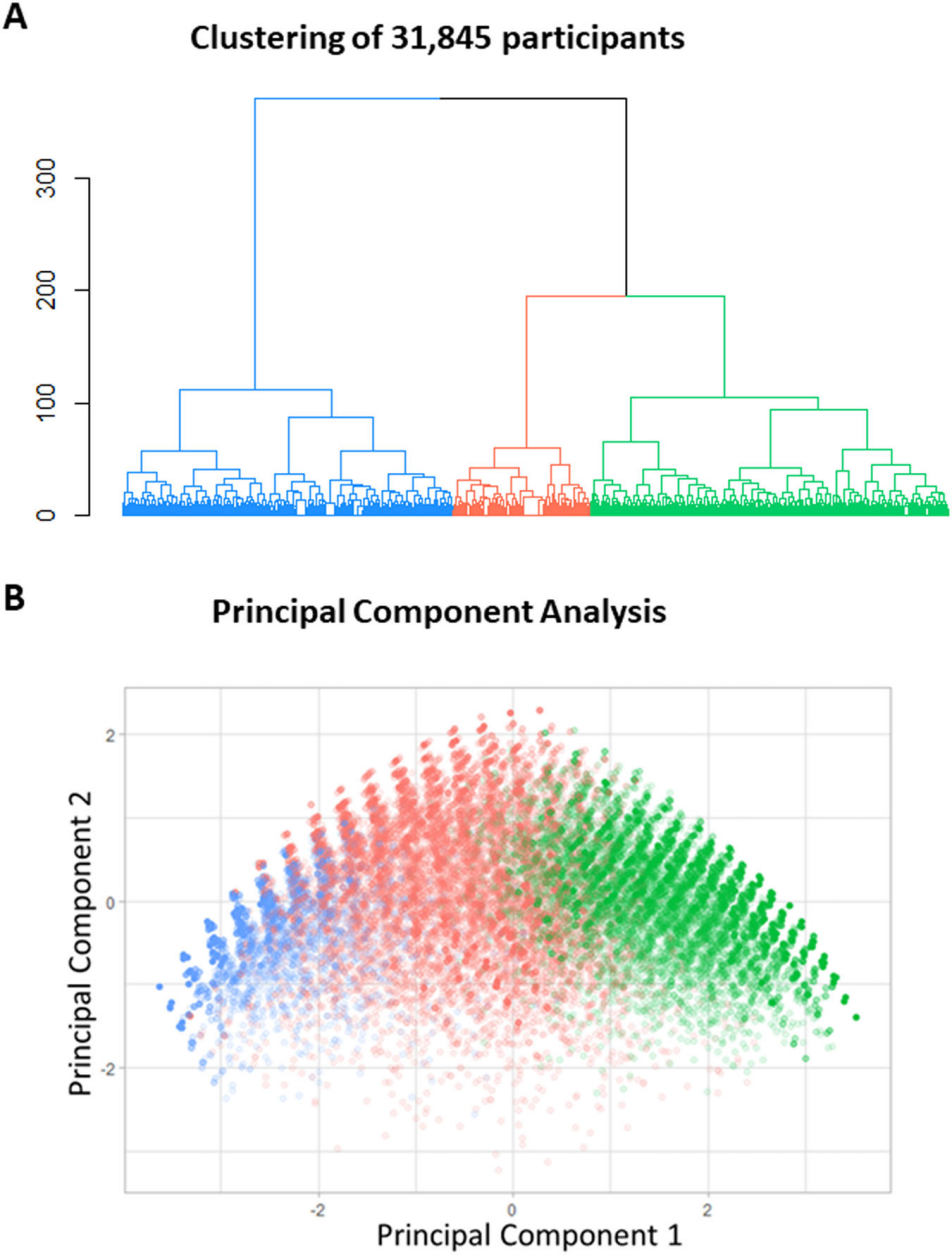
Clustering analysis of 31,845 participants. **A** The dendrogram representing the unsupervised hierarchical clustering of participants. **B** Principal component analysis of participants shows reasonable separation between the three participant clusters. Principal component 1 accounts for 48.2% of the variance in the data. Principal component 2 accounts for 9.1% of the variance in the data.

Using the same set of 14 language comprehension abilities, we performed unsupervised hierarchical cluster analysis of all 31,845 participants. The results of the hierarchical cluster analysis show three robust clusters of participants (Fig. 2A). Again, the distances between three clusters (determined by the height of dendrogram branches) are considerably greater than distances between subclusters, making the three-cluster solution most sensible. The principal component analysis showed reasonable separation between the three participant clusters (Fig. 2B).

The three-cluster solution was stable across multiple seeds as well as consistent across different age groups (4 to 6, 6 to 12, and 12 to 21 years of age, Supplementary Figs. 7 to 9, respectively, the dendrogram shown on the top), across different time points (first evaluation, Supplementary Fig. 10; last evaluation, Fig. 2), and across different evaluation methods (Euclidean distance metric, Fig. 2; Manhattan distance metric, Supplementary Fig. 11; Minkowski distance metric, Supplementary Fig. 12).

**Fig. 3:**
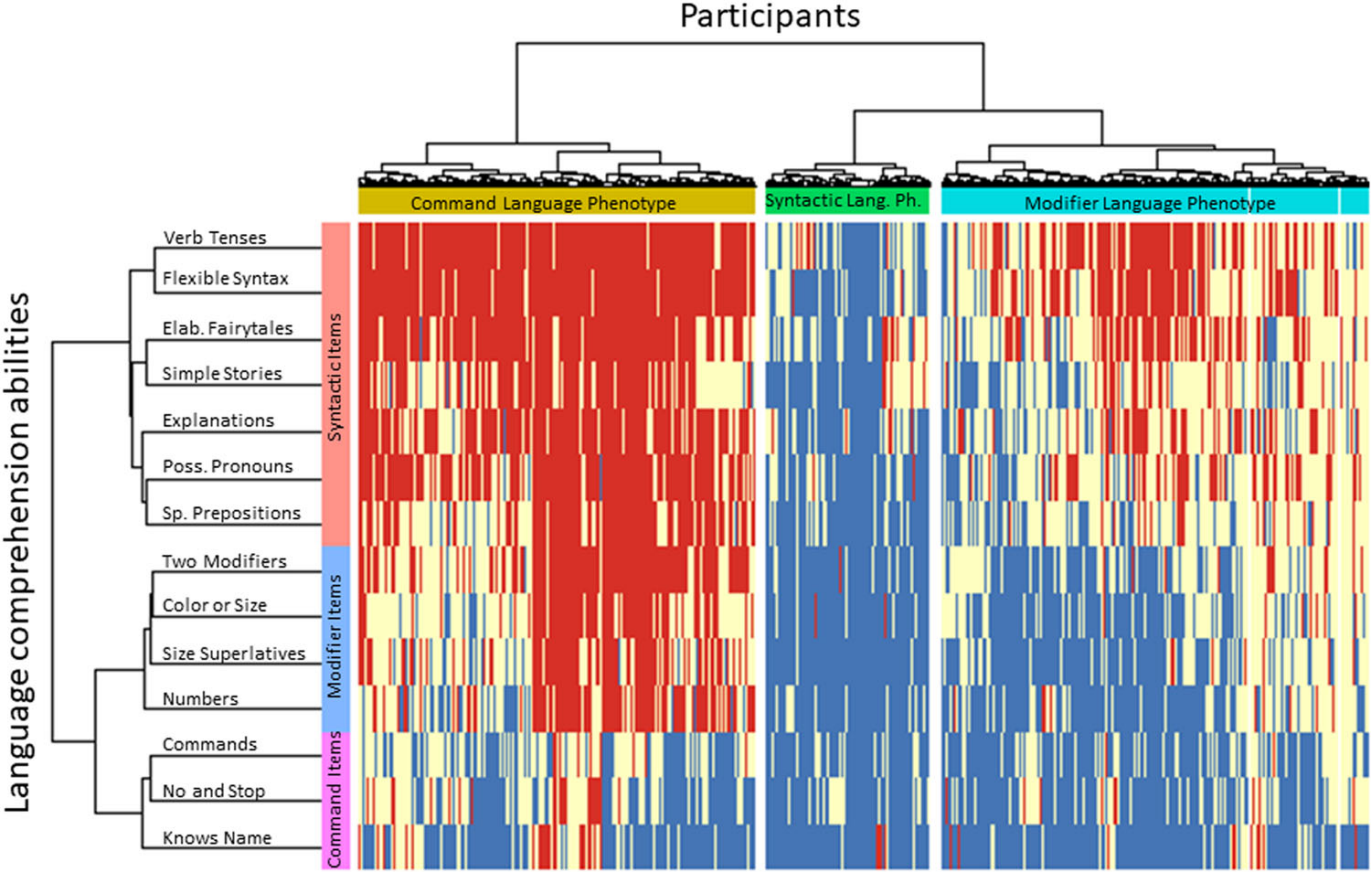
Two dimensional heatmap relating participants to their language comprehension abilities. The 14 language comprehension abilities are shown as rows. The dendrogram representing language comprehension abilities is shown on the left. Participants are shown as 31,845 columns. The dendrogram representing participants is shown on the top. Blue color indicates the presence of a skill (the “very true” answer), red indicates the lack of skill (the “not true” answer), and white indicates the “somewhat true” answer.

The two-dimensional heatmap in Fig. 3 relates participants to their language comprehension abilities: the 14 linguistic abilities are shown as rows and the 31,845 participants are shown as columns. *Blue* stands for the *very true* parent’s response indicating the presence of an ability; *white* stands for the *somewhat true* response; and *red* stands for *not true* response indicating the lack of an ability. The three clusters of participants match the three clusters of linguistic abilities. The narrowest cluster of participants (17%, Table 2) shows the predominant blue color (indicating good skills) across all three clusters of language comprehension abilities and therefore is termed the “Syntactic Language Phenotype.” The biggest cluster of participants (43%) shows the predominant blue color only across command and modifier items and white to red colors across syntactic items, indicating that linguistic abilities of individuals in this cluster are limited to command and modifier comprehension. Accordingly, this cluster of participants was termed the “Modifier Language Phenotype.” The last cluster of participants (40%) shows the predominant blue color only among the command items and white to red colors across syntactic and modifier items, indicating that linguistic abilities of individuals in this cluster are limited to command comprehension. Consequently, this cluster of participants was termed the “Command Language Phenotype.” The close match between the three participant clusters and the three language comprehension abilities clusters confirms the clinical significance of the three-cluster solution.

**Table 2:** Participant cluster statistics

Participant cluster	Number of participants	Percent of total	Age, Mean (SD)	Percent male
Syntactic Language Phenotype	5298	17%	6.3 (2.3)	76%
Modifier Language Phenotype	13,782	43%	6.4 (2.6)	78%
Command Language Phenotype	12,765	40%	6.6 (2.8)	80%
Total	31,845	100%	6.5 (2.6)	78%

### Characterizing and evaluating language comprehension phenotypes

**Fig. 4:**
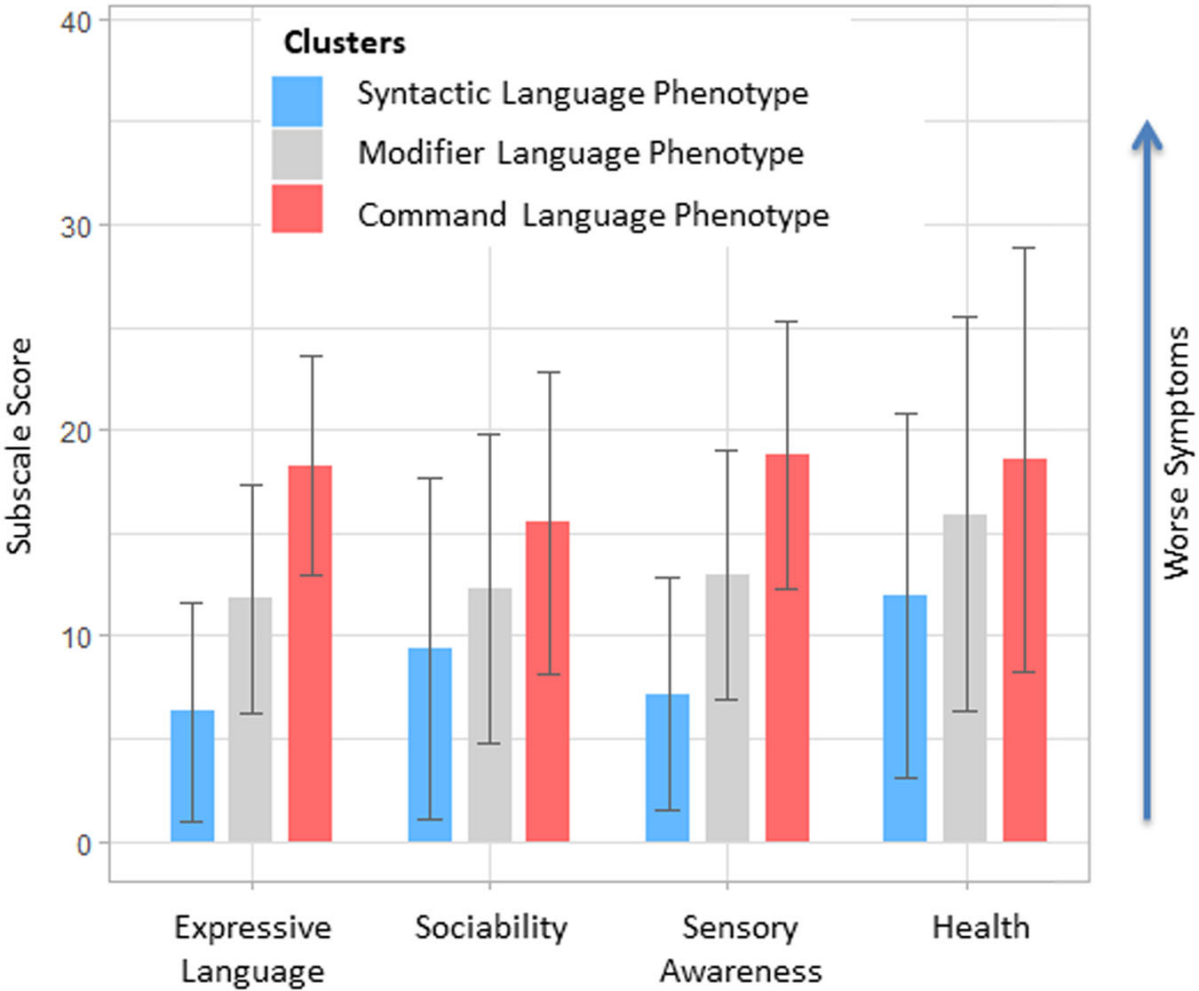
Language comprehension phenotype clusters show significant differences in properties that were not used for clustering, such as expressive language, sociability, sensory awareness, and health. Lower scores indicate milder symptoms. Error bars show standard deviation. All differences between clusters are statistically significant (*t* test: *p* < 0.0001). Supplementary Table 8 summarizes t-test statistics (t, df, and *p*-value).

Participant clusters displayed statistically significant differences in properties that were not used for clustering, such as expressive language, sociability, sensory awareness, and health (two-sample *t* test: *p* < 0.0001) (Fig. 4, Supplementary Table 7), demonstrating that language comprehension phenotypes are associated with symptom severity in individuals with ASD.

The syntactic language phenotype had the greatest proportion of individuals characterized by parents as having mild ASD and the lowest proportion of individuals with severe ASD (Table 3). The command language phenotype had the greatest proportion of individuals characterized by parents as having severe ASD and the lowest proportion of individuals with mild ASD. There was no statistically significant age difference between the three participant clusters (Table 2, Supplementary Fig. 13), suggesting that the differences between the phenotypes were not dependent on participants’ age.

**Table 3:** Occurrence of mild, moderate, and severe ASD parent-reported diagnosis in the three clusters of participants

Participant cluster	Mild ASD	Moderate ASD	Severe ASD
Syntactic Language Phenotype	79%	16%	5%
Modifier Language Phenotype	63%	27%	10%
Command Language Phenotype	39%	33%	28%

There is no common way to define children’s verbal level^24^. In this study, we classified children as *nonverbal* if parents indicated that they were not able “to use 2 words at a time;” and *verbal* when parents indicated that they “can use sentences with 4 or more words.” Other children were defined as *minimally verbal*. Participant clusters displayed significant differences in children’s verbal level: 91% of children in the command language phenotype were nonverbal or minimally verbal, compared to 68% in the modifier language phenotype, and 33% in the syntactic language phenotype (Table 4).

**Table 4:** Verbal level of children in the three clusters of participants

Participant cluster	Verbal	Minimally verbal	Nonverbal
Syntactic Language Phenotype	67.4%	26.6%	6.0%
Modifier Language Phenotype	31.9%	53.1%	15.0%
Command Language Phenotype	8.9%	42.5%	48.6%

Children were classified as *nonverbal* if parents indicated that they were not able “to use 2 words at a time” and *verbal* when parents indicated that they “can use sentences with 4 or more words.” Other children were defined as *minimally verbal*.

## Discussion

The study analyzes 14 language comprehension abilities in 31,845 autistic participants. A three-cluster solution was consistent across a range of ages as well as parameters controlling the unsupervised hierarchical clustering. The first cluster included three abilities (items 1 to 3, Table 1): comprehension of one’s name, responding to ‘No’ or ‘Stop’ and following some commands (Fig. 1A); this cluster of concurrently expressed linguistic abilities was termed “command language.” The second cluster included four abilities (items 4 to 7): comprehension of simple color/size modifiers, understanding of several modifiers in a sentence, understanding of size superlatives, and number comprehension; this cluster of co-expressed abilities was termed “modifier language.” The third cluster included the remaining 7 abilities (items 8 to 14): comprehension of spatial prepositions, verb tenses, flexible syntax, possessive pronouns, explanations, simple stories, and elaborate fairytales; this cluster of co-expressed abilities was termed “syntactic language.” Reassuringly, principal component analysis (Fig. 1B) and correlation analysis (Supplementary Fig. 2) corroborated the three-cluster solution. This arrangement of language comprehension items into the three clusters was stable across different evaluation methods (Supplementary Fig. 1), different age groups (Supplementary Figs. 3 to 5), and different time points (first evaluation, Supplementary Fig. 6; last evaluation, Fig. 1). While the choice of cluster names can be critiqued, the existence of three distinct clusters of co-expressed abilities is self-evident from Fig. 1. Critically, there was no design or hypothesis involved in the classification process; both analysis techniques – unsupervised hierarchical clustering and principal component analysis – were completely data-driven.

Independently, unsupervised hierarchical cluster analysis assigned 31,845 participants to clusters. The three-cluster solution was consistent across a range of ages as well as parameters controlling the unsupervised hierarchical clustering (Fig. 2). The two-dimensional heatmap analysis related participants’ clusters (Fig. 3, the dendrogram shown on the top) to language comprehension abilities clusters (the dendrogram shown on the left). Participants in the syntactic language phenotype cluster acquired most language comprehension abilities tested by the 14 items (indicated by the predominant blue color across all items). Participants in the modifier language phenotype cluster acquired most command and modifier language abilities (indicated by the blue color) and did not acquire most syntactic language abilities (indicated by the red and white colors). Participants in the command language phenotype cluster acquired command language abilities (indicated by the blue color) and did not acquire syntactic and modifier language abilities (indicated by the predominant red color).

One of several possible explanations for differences in language comprehension could be the differential cultural exposure of participants to linguistic concepts. For example, if participants were never exposed to numbers, they would not understand the concept of numbers; if they were never exposed to spatial prepositions, they would not understand spatial prepositions. However, by four years of age (the lower cutoff in our study) participants were exposed to a variety of items listed in Table 1^3^. Furthermore, limiting cluster analysis to older participants 6 to 12 and 12 to 21 years of age also demonstrated the same arrangement of language comprehension abilities into the three clusters (compare Supplementary Figs. 3–5) and the same three language comprehension phenotype clusters (compare Supplementary Figs. 7–9). Therefore, differential exposure cannot explain the observed three language comprehension phenotypes. Accordingly, the three language comprehension phenotypes can only be explained by some common regulatory mechanisms. For some reason, individuals in the modifier language phenotype were not able to acquire the mechanism underlying syntactic skills, and individuals in the command language phenotype were not able to acquire both mechanisms underlying modifier and syntactic skills. Taken together with cluster analysis of linguistic abilities (Fig. 1), on the simplest level, these results suggest a common regulatory mechanism behind comprehension of spatial prepositions, verb tenses, flexible syntax, possessive pronouns, explanations, simple stories, and elaborate fairytales; a different mechanism behind color, size, and number modifiers comprehension; and still a different mechanism behind commands comprehension. Elucidation of possible mechanisms underlying syntactic, modifier, and command language phenotypes is scientifically interesting and may be important for developing better treatment options for individuals with language deficits^25^.

Language comprehension phenotypes displayed significant differences in properties that were not used for clustering, such as expressive language, sociability, sensory awareness, and health, demonstrating that language comprehension phenotypes are associated with symptom severity in individuals with ASD (Fig. 4).

As expected, language comprehension phenotypes were associated with individuals’ expressive language level: the command language phenotype had the greatest proportion of nonverbal and minimally verbal individuals (91%), followed by the modifier language phenotype that included 68% of nonverbal and minimally verbal individuals, followed by syntactic language phenotype that had 33% of nonverbal and minimally verbal individuals (Table 4). However, similar to previous reports, this association was not absolute^13,26,27^. A mix of verbal abilities was observed in each language phenotype, suggesting that language comprehension and language production are clinically different phenomena. This finding points to a possibility of improvement of the current terminology that primarily describes an individual’s communication level in terms of their use of spoken language: *nonverbal*, *minimally verbal*, and *verbal*. Arguably, an individual’s communication level is defined to a greater extent by their language comprehension phenotype than by their verbal ability; for example, a *nonverbal* individual with syntactic language phenotype may have typical ability to communicate albeit nonverbally, while a *verbal* person with command language phenotype does not have a typical ability to communicate by any means.

Studies of app users provide access to a large number of individuals, but have obvious downsides, such as relying on parent reports. On one hand, parents may yield to wishful thinking and overestimate their children’s abilities^28^; on the other, parents possess deep understanding of their children. This understanding may be especially important for language comprehension assessment, which evaluation in a clinical office can be very tricky. Additionally, multiple previous studies suggest that parent reports of language skills do not significantly differ from direct clinicians’ assessments^29,30^ and studies of our database also indicate consistent and accurate parent reports^13,31,32^. Another concern is conducting this study in a predominantly young population. Children are still learning language and therefore their language phenotype could be transitional. Future research should confirm the existence of the three language comprehension phenotypes in a greater number of older individuals with ASD. Finally, caregivers were not asked about hearing or other sensory impairments known to be associated with language development and therefore we could not exclude those individuals from the study. Additionally, caregivers were not asked about intellectual disability and therefore we could not analyze it in relation to phenotypes.

The study confirms previous reports that language comprehension does not always coincide with expressive language level^13,26,27^. In this study 41% of verbal individuals have been clustered outside of the most-advanced syntactic language comprehension phenotype. Furthermore, 33% of individuals in the most-advanced syntactic language comprehension phenotype were nonverbal or minimally verbal. One-dimensional description of individuals as *verbal*, *minimally verbal*, or *nonverbal* is not fully reporting an individual’s communication ability. Identification of three distinct language comprehension phenotypes in autistic individuals provides an opportunity to improve classification of individuals’ communication level. A two-dimensional classification in terms of verbal abilities and language-comprehension-level provides a more precise description of an individual’s communication ability.

Language-comprehension-level classification will be aided by developing new assessments capable of testing syntactic language comprehension at the level of 2.5 to 4.5-year-old typical children and reporting the results in terms of a language comprehension phenotype (command, modifier, or syntactic). Existing tests for syntactic language comprehension – the Preschool Language Scales (PLS-5)^33^, Clinical Evaluation of Language Fundamentals (CELF-5)^34^, and the Test for Reception Of Grammar (TROG)^35^ – are geared for children 4.5 years of age and older^3^. They use overly long instructions and combine multiple grammatical forms that are beyond the working memory resources of younger children. The ongoing work includes the development of both clinician-^36,37^ and parent-report^3,12,32,38^ assessments. Using assessments of language comprehension phenotype to measure language acquisition will help focus language therapy on this important aspect of language development and may improve outcomes in individuals with ASD.

## Methods

### Participants

Participants were children and adolescents using a language therapy app that was made available gratis at all major app stores in September 2015^14,39–42^. Once the app was downloaded, caregivers were asked to register and to provide demographic details, including the child’s diagnosis and age. Caregivers consented to anonymized data analysis and completed the Autism Treatment Evaluation Checklist (ATEC)^43^, and an evaluation of language comprehension using the Mental Synthesis Evaluation Checklist (MSEC)^12^. Inclusion criteria were as follows: parent-reported ASD diagnosis (in order to map language comprehension phenotypes onto existing autism subtypes), absence of seizures (that commonly result in intermittent, unstable language comprehension deficits^44^), absence of serious and moderate sleep problems (that are also associated with intermittent, unstable language comprehension deficits^31^), age range of 4–21 years (the lower age cutoff was chosen to ensure that participants were exposed to all variety of items listed in Table 1^3^; the upper age cutoff was chosen to avoid analysis of participants who may be linguistically declining due to aging). When caregivers have completed several evaluations, the last evaluation was used for analysis. Autism level (mild/Level 1, moderate/Level 2, or severe/Level 3) was reported by caregivers. Pervasive Developmental Disorder and Asperger Syndrome were combined with mild autism for analysis as recommended by DSM-5^1^. A good reliability of such parent-reported diagnosis has been previously demonstrated^32^. The study included 31,845 participants, the average age was 6.5 ± 2.6 years (range of 4 to 21 years), 78% participants were males. The education level of participants’ parents was the following: 92% with at least a high school diploma, 71% with at least college education, 38% with at least a master’s, and 6% with a doctorate. Most participants were English-speakers (46%), followed by Spanish-speakers (27%), Portuguese-Speakers (10%), and Italian-speakers (6%). Most participants resided in the USA (41%), Brazile (9%), Mexico (5%), and Italy (5%). All caregivers consented to anonymized data analysis and publication of the results. The study was conducted in compliance with the Declaration of Helsinki^45^. Using the Department of Health and Human Services regulations found at 45 CFR 46.101(b)(4), the Biomedical Research Alliance of New York LLC Institutional Review Board (IRB) determined that this research project is exempt from IRB oversight.

### Evaluations

As a part of ATEC^43^ and MSEC^3,12–14,31,32^ subscales, parents responded to fourteen language comprehension questions described in Table 1 (mean and standard deviations for each item are shown in Supplementary Table 1). Additionally, caregiver responses to the ATEC^43^ questionnaire were used to assess children’s expressive language, sociability, sensory awareness, and health (Tables S2-S6). Various studies confirmed validity and reliability of ATEC^46,47^ and several trials confirmed ATEC’s ability to measure longitudinal changes^48–51^. Whitehouse et al. used ATEC as a primary outcome measure for a randomized controlled trial of their iPad-based intervention for ASD named TOBY and noted ATEC’s “internal consistency and adequate predictive validity”^52^. ATEC total score and the four subscale measures demonstrated a strong correlation with the CARS score^46,53^. A lower score in all subscales indicates milder symptoms.

All available *language comprehension* items from ATEC and MSEC (i.e., items 1, 2, 3 from ATEC subscale 1, Supplementary Table 2, and items 1, 2, 6, 7, 8, 9, 10, 11, 12, 13, 20 from MSEC, Supplementary Table 6) were included in the cluster analysis (Table 1).

Parents’ answers on the ATEC expressive language subscale were used to determine children’s verbal level. Children were classified as *nonverbal* if parents indicated that they were not able “to use 2 words at a time.” Children were classified as *verbal* when parents indicated that they “can use sentences with 4 or more words.” Other children were defined as *minimally verbal*.

### Statistics and reproducibility

Unsupervised hierarchical clustering was performed using Ward’s agglomeration method with a Euclidean distance metric. Two-dimensional heatmap was generated using the “pheatmap” package of R, freely available language for statistical computing.

### Supplementary information

Supplementary Information

## Data Availability

De-identified raw data from this manuscript are available from the corresponding author upon reasonable request.

## Code availability

Code is available from the corresponding author upon reasonable request.
