# Supplementary material for: Are there distinct levels of language comprehension in autistic individuals – cluster analysis
Source: Npj Ment Health Res. 2024 Apr 10;3:19. doi: 10.1038/s44184-024-00062-1 (PMC11006660; doi:10.1038/s44184-024-00062-1)
Supplement: Supplementary file 1 — Supplementary Information [file 44184_2024_62_MOESM1_ESM.pdf]

Supplementary Material

Supplementary Table 1. Language comprehension items as they were posed to parents and their mean and standard deviation. A lower score indicates better language comprehension ability. The answers choices were: very true (0 points), somewhat true (1 point), and not true (2 points).

	**Language comprehension items (verbatim)**	**Mean(SD)**
1	Knows own name	0.31(0.59)
2	Responds to ‘No’ or ‘Stop’	0.47(0.61)
3	Can follow some commands	0.44(0.56)
4	Understands some simple modifiers (i.e., green apple vs. red apple or big apple vs. small apple)	0.76(0.75)
5	Understands several modifiers in a sentence (i.e., small green apple)	0.94(0.78)
6	Understands size (can select the largest/smallest object out of a collection of objects)	0.75(0.74)
7	Understands NUMBERS (i.e., two apples vs. three apples)	0.77(0.77)
8	Understands spatial prepositions (i.e., put the apple ON TOP of the box vs. INSIDE the box vs. BEHIND the box)	1.01(0.72)
9	Understands verb tenses (i.e., I will eat an apple vs. I ate an apple)	1.49(0.68)
10	Understands simple stories that are read aloud	1.07(0.72)
11	Understands elaborate fairytales that are read aloud (i.e., stories describing FANTASY creatures)	1.39(0.71)
12	Understands possessive pronouns (i.e., your apple vs. her apple)	1.22(0.75)
13	Understands the change in meaning when the order of words is changed (i.e., understands the difference between 'a cat ate a mouse' vs. 'a mouse ate a cat')	1.44(0.72)
14	Understands explanations about people, objects or situations beyond the immediate surroundings (e.g., “Mom is walking the dog,” “The snow has turned to water”).	1.23(0.74)

Supplementary Table 2: ATEC subscale 1: Speech/Language/Communication. The answers choices were: very true (0 points), somewhat true (1 point), and not true (2 points).

1. Knows own name
2. Responds to ‘No’ or ‘Stop’
3. Can follow some commands
4. Can use one word at a time (No!, Eat, Water, etc.)
5. Can use 2 words at a time (Don't want, Go home)
6. Can use 3 words at a time (Want more milk)
7. Knows 10 or more words
8. Can use sentences with 4 or more words
9. Explains what he/she wants
10. Asks meaningful questions
11. Speech tends to be meaningful/relevant
12. Often uses several successive sentences
13. Carries on fairly good conversation

14. Has normal ability to communicate for his/her age

Supplementary Table 3: ATEC subscale 2: Sociability. The answers choices were: very true (0 points), somewhat true (1 point), and not true (2 points).

1. Seems to be in a shell – you cannot reach him/her
2. Ignores other people
3. Pays little or no attention when addressed
4. Uncooperative and resistant
5. No eye contact
6. Prefers to be left alone
7. Shows no affection
8. Fails to greet parents
9. Avoids contact with others
10. Does not imitate
11. Dislikes being held/cuddled
12. Does not share or show
13. Does not wave 'bye bye'
14. Disagreeable/not compliant
15. Temper tantrums
16. Lacks friends/companions
17. Rarely smiles
18. Insensitive to other's feelings
19. Indifferent to being liked
20. Indifferent if parent(s) leave

Supplementary Table 4: ATEC subscale 3: Sensory/Cognitive awareness. The answers choices were: very true (0 points), somewhat true (1 point), and not true (2 points).

1. Responds to own name
2. Responds to praise
3. Looks at people and animals
4. Looks at pictures (and T.V.)
5. Does drawing, coloring, art
6. Plays with toys appropriately
7. Appropriate facial expression
8. Understands stories on T.V.
9. Understands explanations
10. Aware of environment
11. Aware of danger
12. Shows imagination
13. Initiates activities
14. Dresses self
15. Curious, interested
16. Venturesome – explores
17. "Tuned in" — Not spacey
18. Looks where others are looking

Supplementary Table 5: ATEC subscale 4: Health/Physical/Behavior. The answers choices were: not a problem (0 points), minor problem (1 point), moderate problem (2 points), and serious problem (3 points).

1. Bed-wetting
2. Wets pants/diapers
3. Soils pants/diapers
4. Diarrhea
5. Constipation
6. Sleep problems
7. Eats too much/too little
8. Extremely limited diet
9. Hyperactive
10. Lethargic
11. Hits or injures self
12. Hits or injures others
13. Destructive
14. Sound-sensitive
15. Anxious/fearful
16. Unhappy/crying
17. Seizures
18. Obsessive speech
19. Rigid routines
20. Shouts or screams
21. Demands sameness
22. Often agitated
23. Not sensitive to pain
24. “Hooked” or fixated on certain objects/topics
25. Repetitive movements (stimming, rocking, etc.)

Supplementary Table 6: MSEC subscale. The answers choices were: very true (0 points), somewhat true (1 point), and not true (2 points). All MSEC language comprehension items (1,2,6,7,8,9,10,11,12,13,20) were included in cluster analysis.

1. Understands simple stories that are read aloud
2. Understands elaborate fairy tales that are read aloud (i.e. stories describing FANTASY creatures)
3. Draws a VARIETY of RECOGNIZABLE images (objects, people, animals, etc.)
4. Can draw a NOVEL image following YOUR description (e.g. a three-headed horse)
5. Engages in a VARIETY of make-believe activities (such as: playing house, playing with toy soldiers, building forts and castles, etc.)
6. Understands some simple modifiers (i.e. green apple vs. red apple or big apple vs. small apple)
7. Understands several modifiers in a sentence (i.e. small green apple)
8. Understands size (can select the largest/smallest object out of a collection of objects)
9. Understands possessive pronouns (i.e. your apple vs. her apple)
10. Understands spatial prepositions (i.e. put the apple ON TOP of the box vs. INSIDE the box vs. BEHIND the box)
11. Understands verb tenses (i.e. I will eat an apple vs. I ate an apple)
12. Understands the change in meaning when the order of words is changed (i.e. understands the difference between 'a cat ate a mouse' vs. 'a mouse ate a cat')
13. Understands NUMBERS (i.e. two apples vs. three apples)
14. Can perform simple arithmetic: 2 + 3 = ?

15. Can add larger numbers: 7 + 6 = ?
16. Can perform simple subtraction: 3 – 2 = ?
17. Can subtract larger numbers: 15 – 7 = ?
18. Can perform simple multiplication: 2 × 2 = ?
19. Can multiply larger numbers: 6 × 7 = ?
20. Understands explanations about people, objects or situations beyond the immediate surroundings (e.g., “Mom is walking the dog,” “The snow has turned to water”)

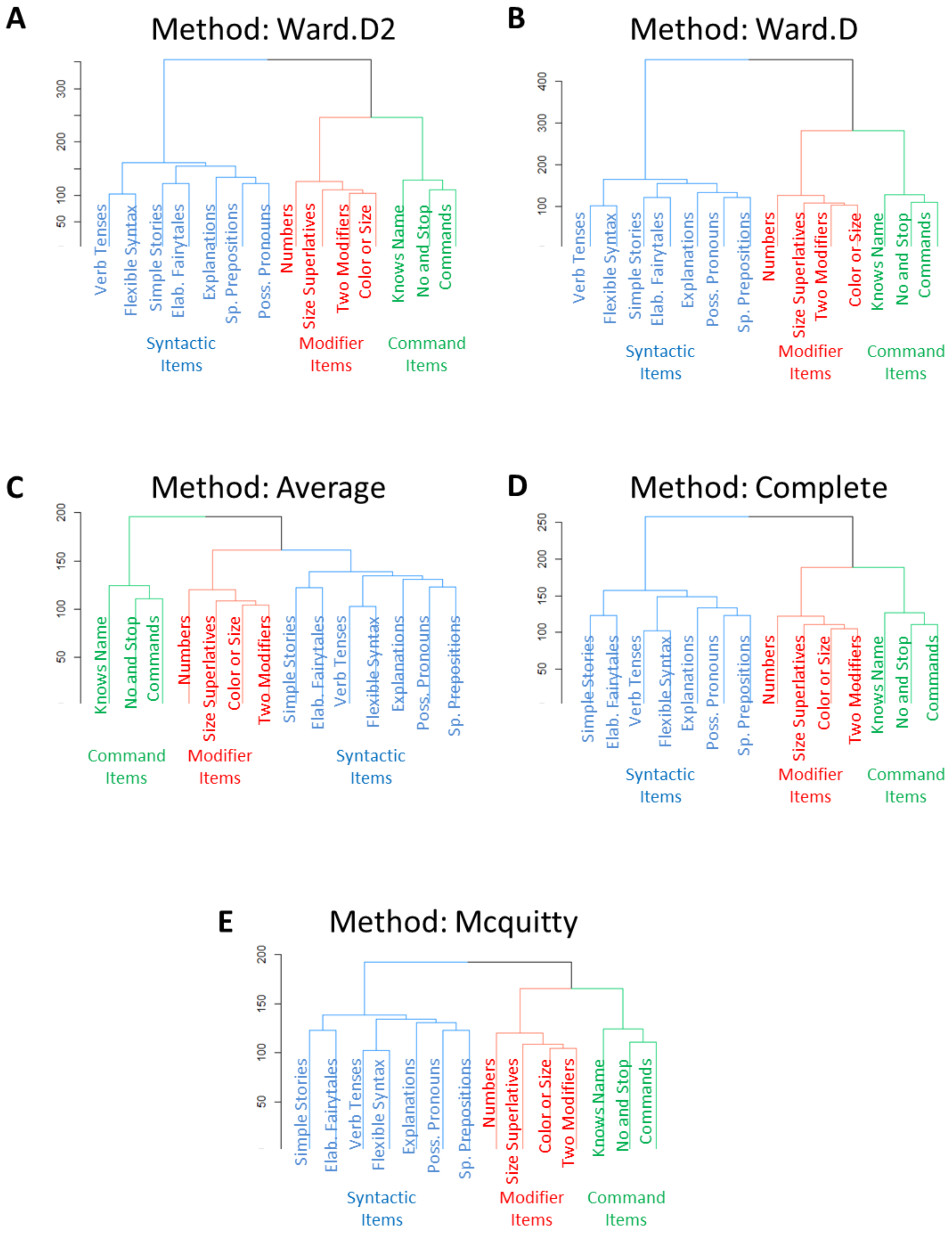

Supplementary Figure 1. The three-cluster solution of the 14 language comprehension abilities was

stable across different evaluation methods. A: Ward.D2. B: Ward.D. C: Average. D: Complete. E: Mcquitty.

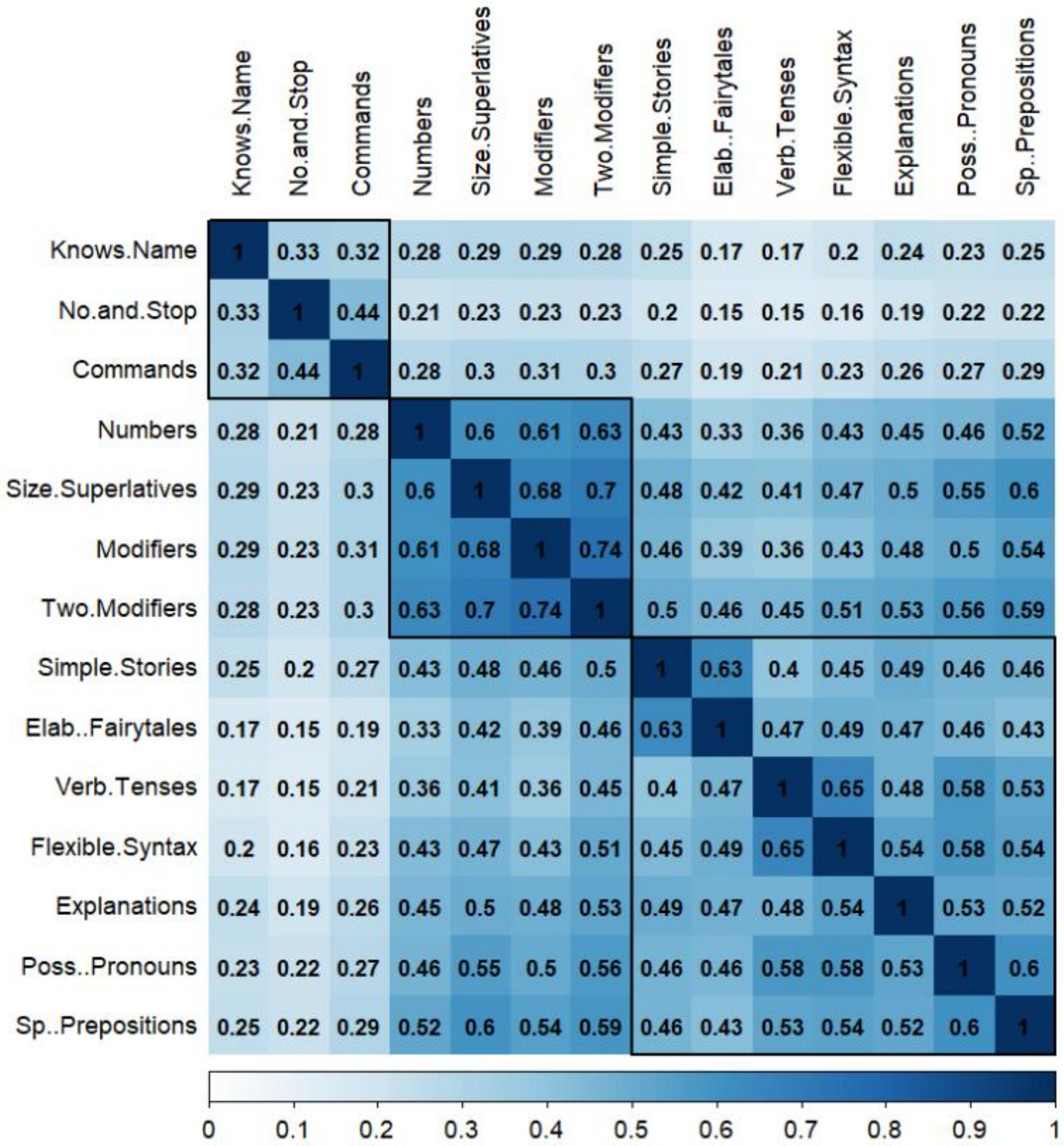

Supplementary Figure 2. Spearman's correlation between 14 language comprehension abilities ($p < 0.0001$). The three clusters of abilities identified by the unsupervised hierarchical cluster analysis are indicated with black squares. Language comprehension abilities shows stronger correlation between items within each of the three clusters and weaker correlation between items outside each cluster.

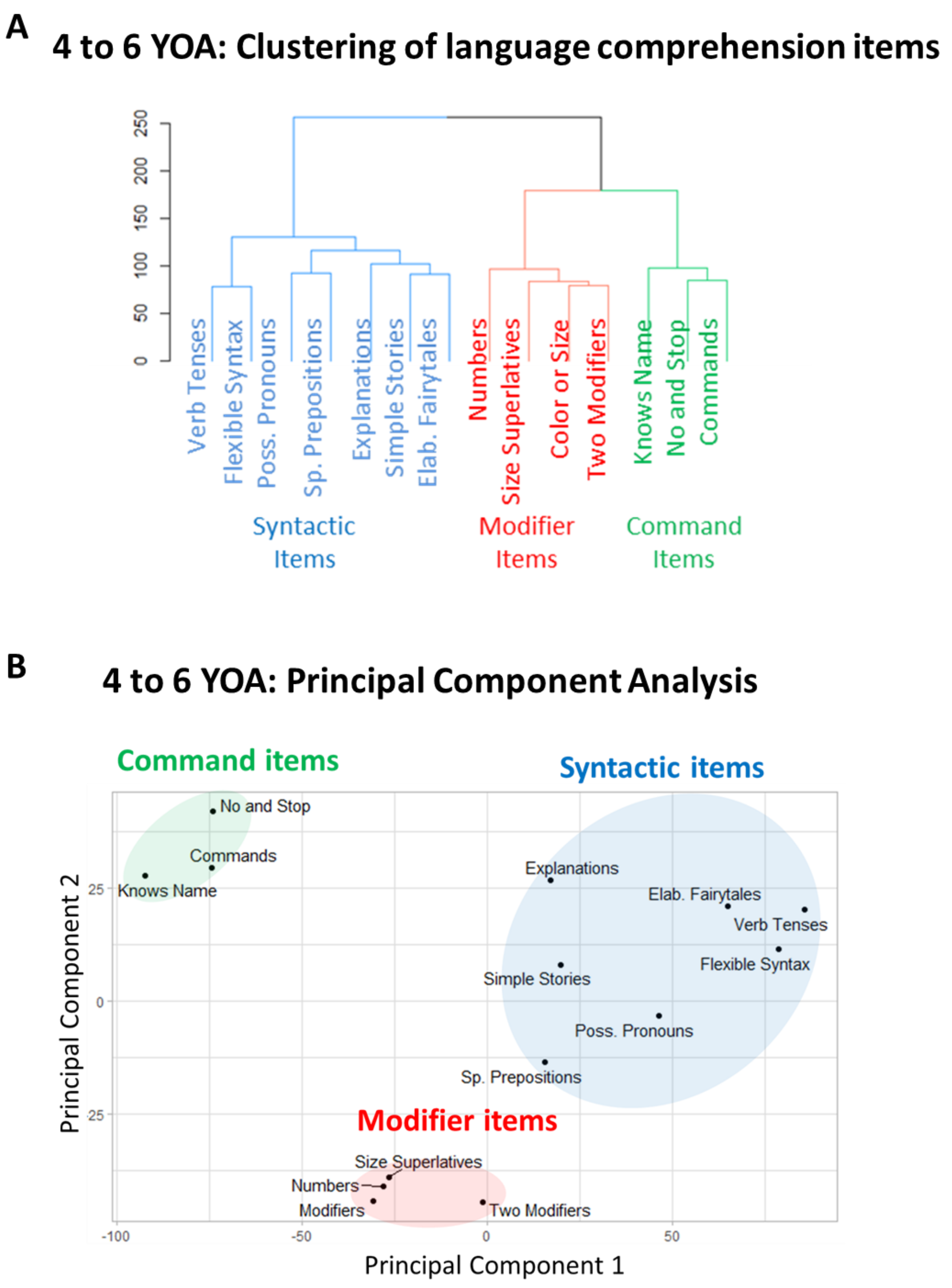

Supplementary Figure 3. Clustering analysis of language comprehension items limited to 17,940 participants (4 to 6 years of age. (A) The dendrogram representing the unsupervised hierarchical clustering of language comprehension abilities. (B) Principal component analysis of the 14 language comprehension abilities shows clear separation between command, modifier, and prepositional items. Principal component 1 accounts for 42.6% of the variance in the data. Principal component 2 accounts for 12.4% of the variance in the data.

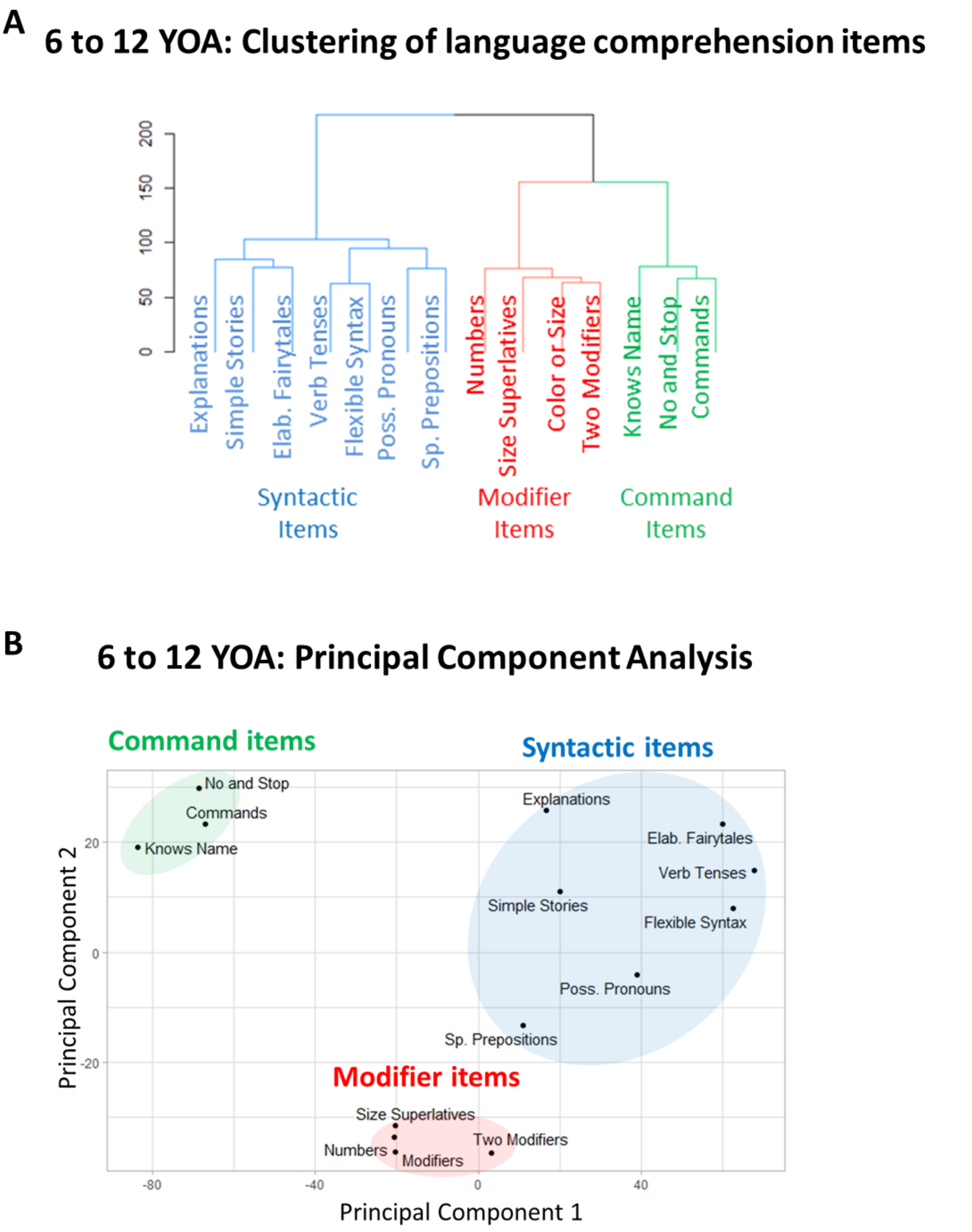

Supplementary Figure 4. Clustering analysis of language comprehension items limited to 12,301 participants (6 to 12 years of age). (A) The dendrogram representing the unsupervised hierarchical clustering of language comprehension abilities. (B) Principal component analysis of the 14 language comprehension abilities shows clear separation between command, modifier, and prepositional items. Principal component 1 accounts for 45.8% of the variance in the data. Principal component 2 accounts for 12.0% of the variance in the data.

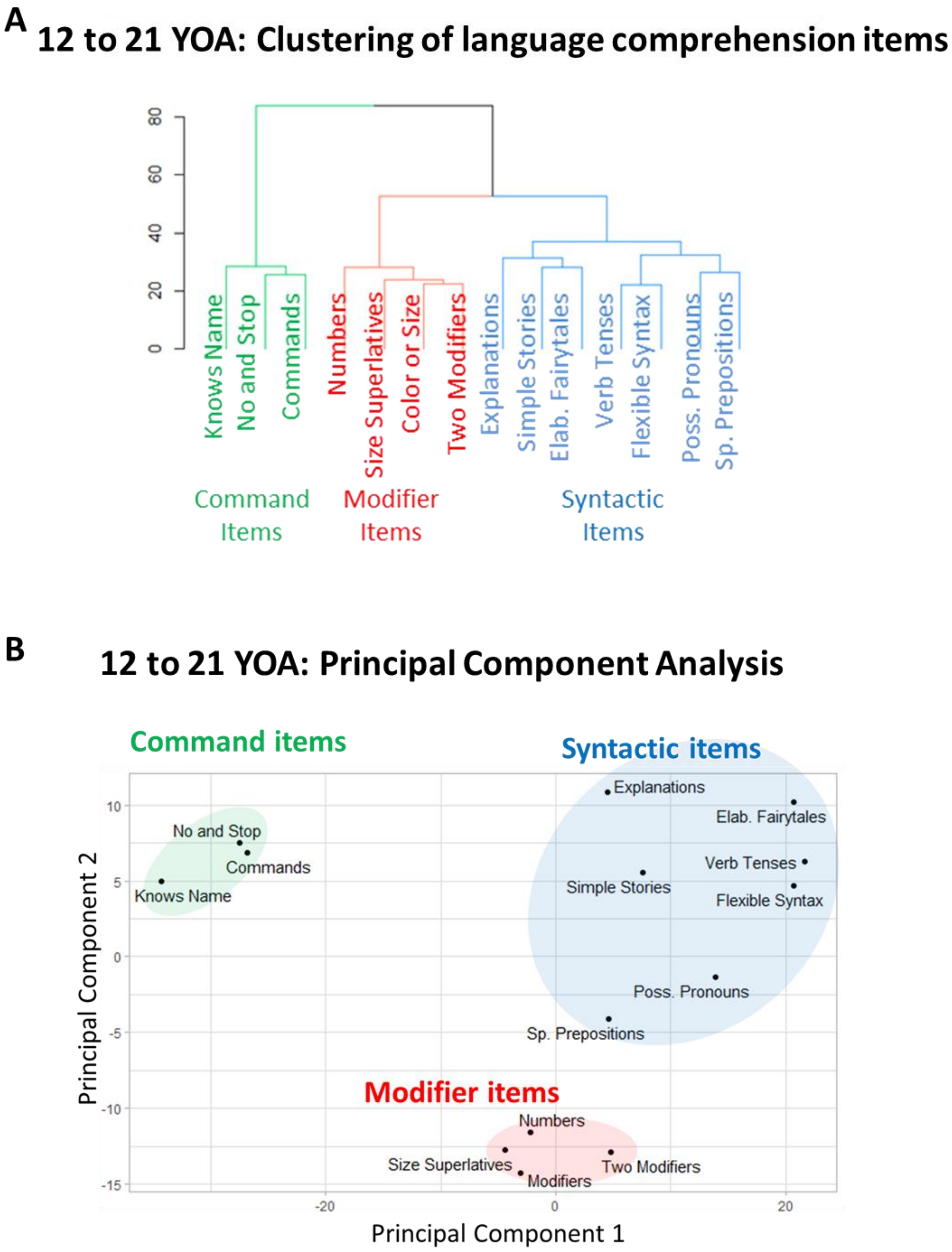

Supplementary Figure 5. Clustering analysis of language comprehension items limited to 1,604 participants (12 to 21 years of age). (A) The dendrogram representing the unsupervised hierarchical clustering of language comprehension abilities. (B) Principal component analysis of the 15 language comprehension abilities shows clear separation between command, modifier, and prepositional items.

Principal component 1 accounts for 46.6% of the variance in the data. Principal component 2 accounts for 12.2% of the variance in the data.

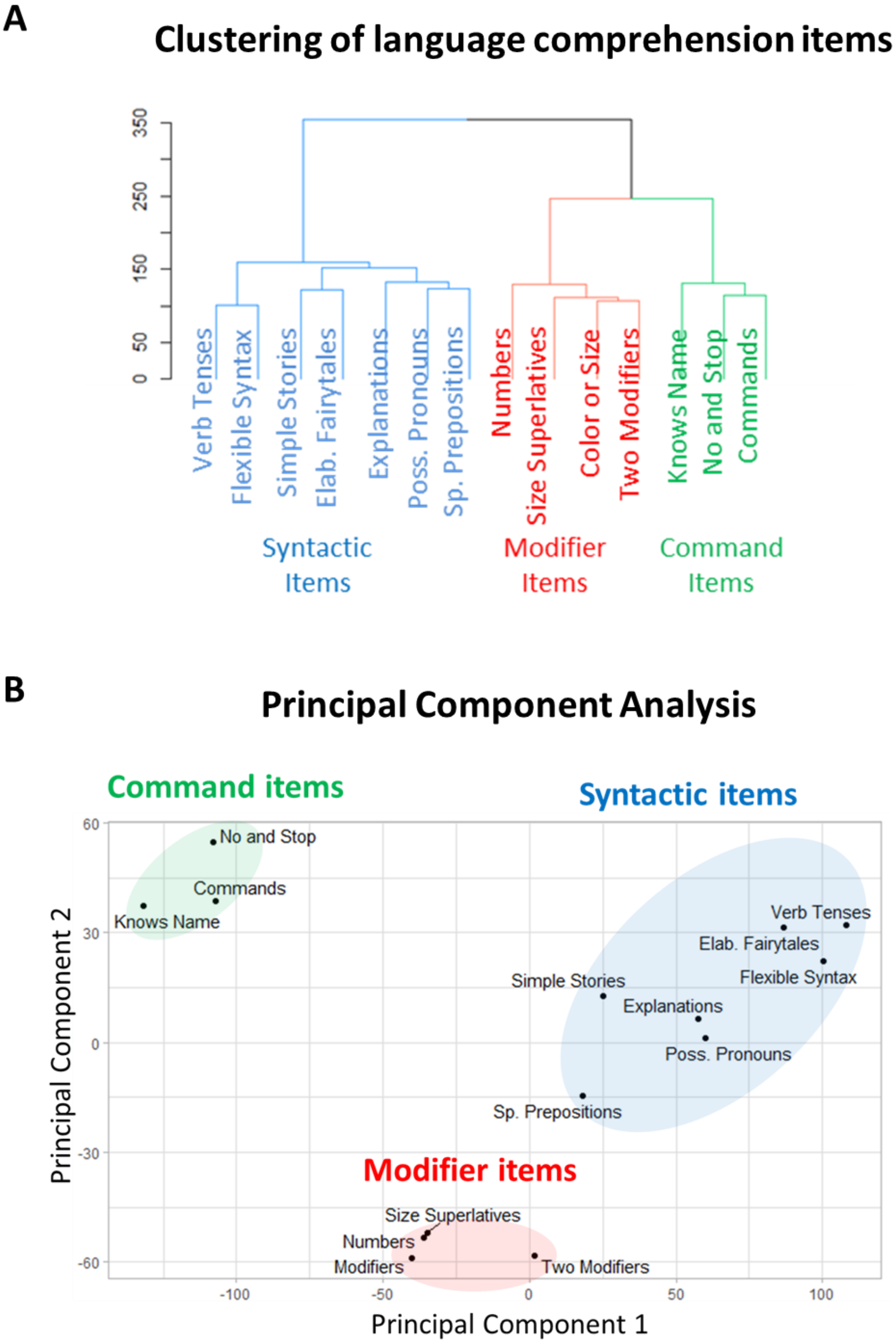

Supplementary Figure 6. Clustering analysis of language comprehension items (31,845 participants, 4 to 21 years of age, first evaluation). (A) The dendrogram representing the unsupervised hierarchical clustering of language comprehension abilities. (B) Principal component analysis of the 14 language comprehension abilities shows clear separation between command, modifier, and prepositional items.

Principal component 1 accounts for 44.9% of the variance in the data. Principal component 2 accounts for 11.6% of the variance in the data.

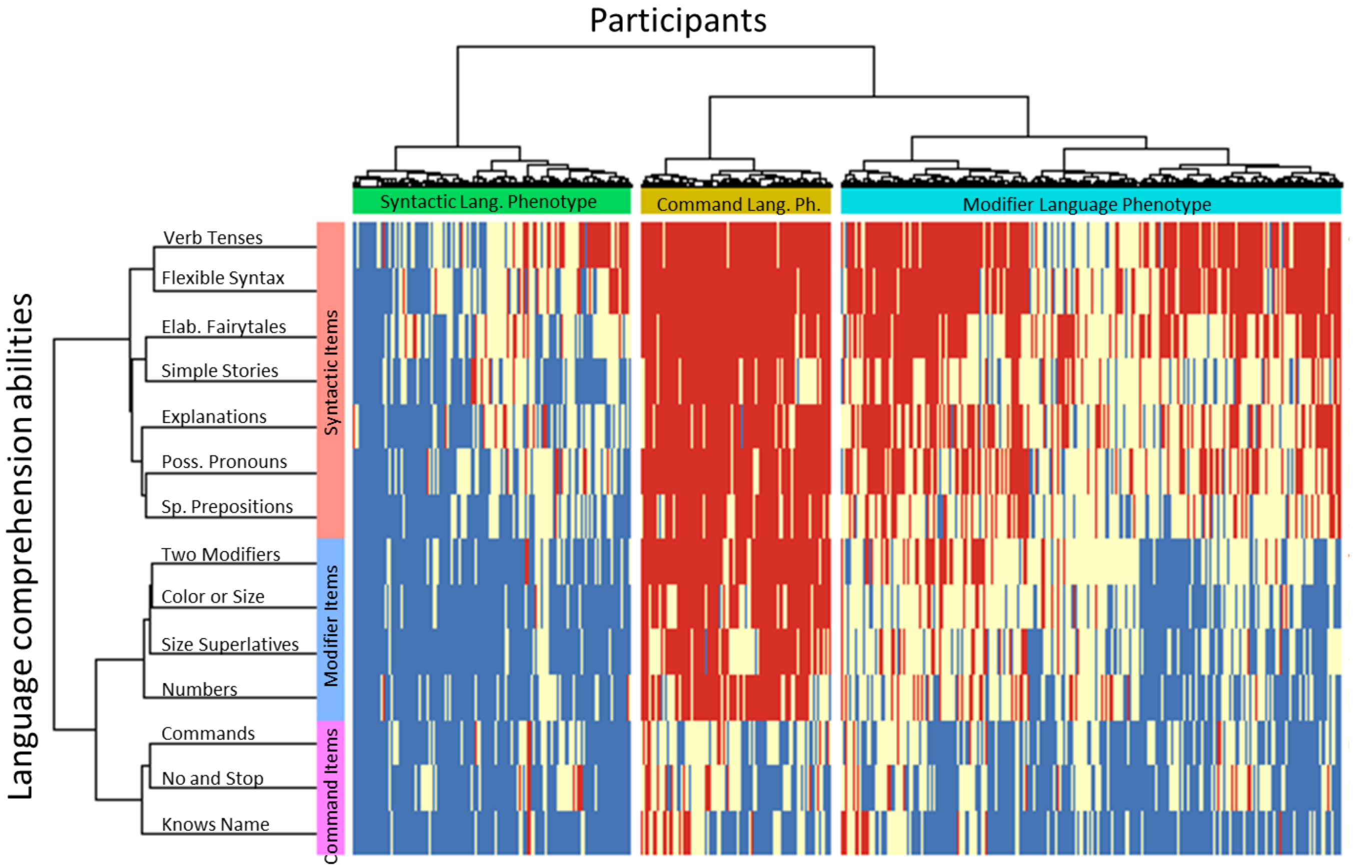

Supplementary Figure 7. Two dimensional heatmap relating 17,940 participants (**of 4 to 6 years of age**) to their language comprehension abilities. The 14 language comprehension abilities are shown as rows. The dendrogram representing language comprehension abilities is shown on the left. Participants are shown as 17,940 columns. The dendrogram representing participants is shown on the top. Blue color indicates the presence of a skill (the "very true" answer), red indicates the lack of skill (the "not true" answer), and white indicates the "somewhat true" answer.

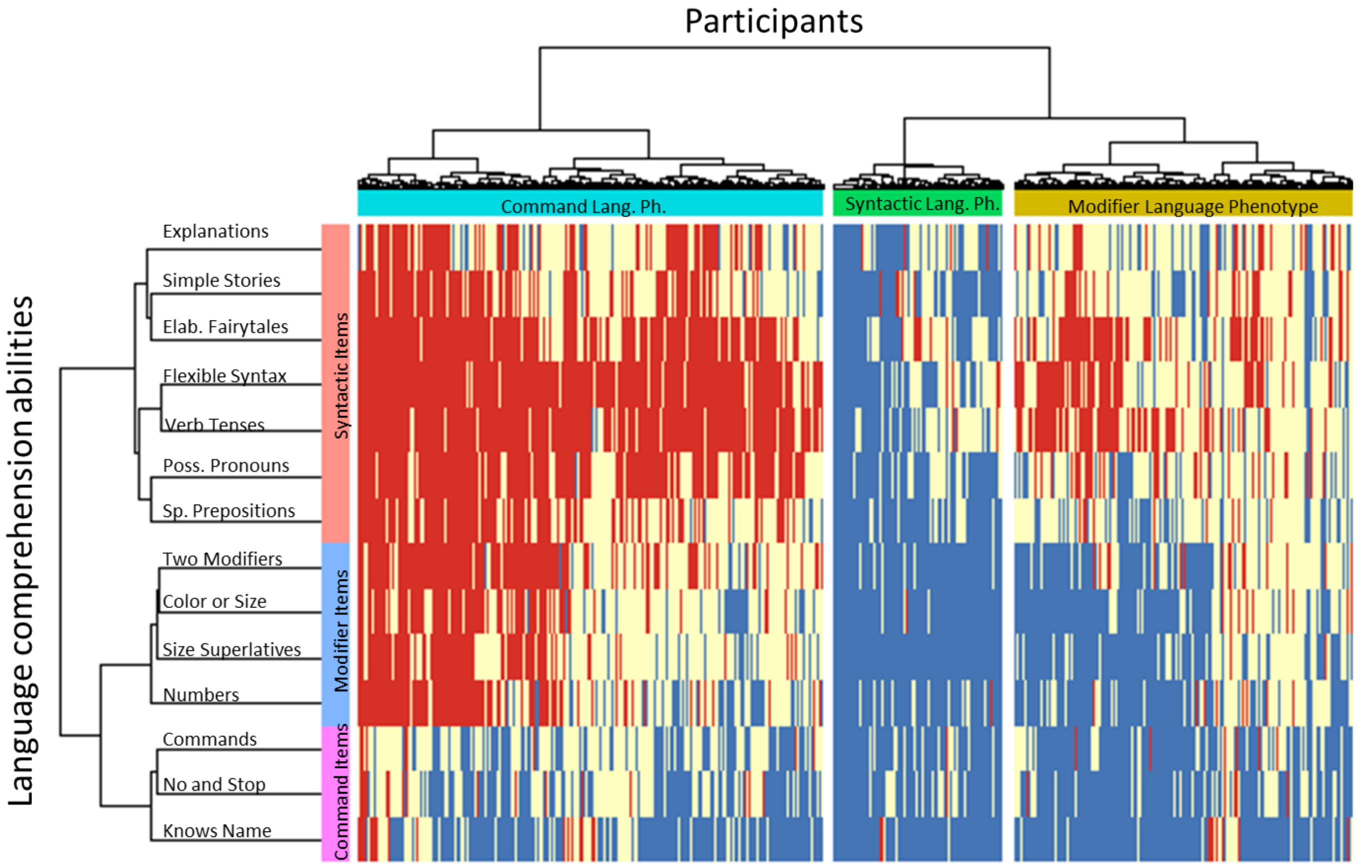

Supplementary Figure 8. Two dimensional heatmap relating 12,301 participants (**of 6 to 12 years of age**) to their language comprehension abilities. The 14 language comprehension abilities are shown as rows. The dendrogram representing language comprehension abilities is shown on the left. Participants are shown as 12,301 columns. The dendrogram representing participants is shown on the top. Blue color indicates the presence of a skill (the “very true” answer), red indicates the lack of skill (the “not true” answer), and white indicates the “somewhat true” answer.

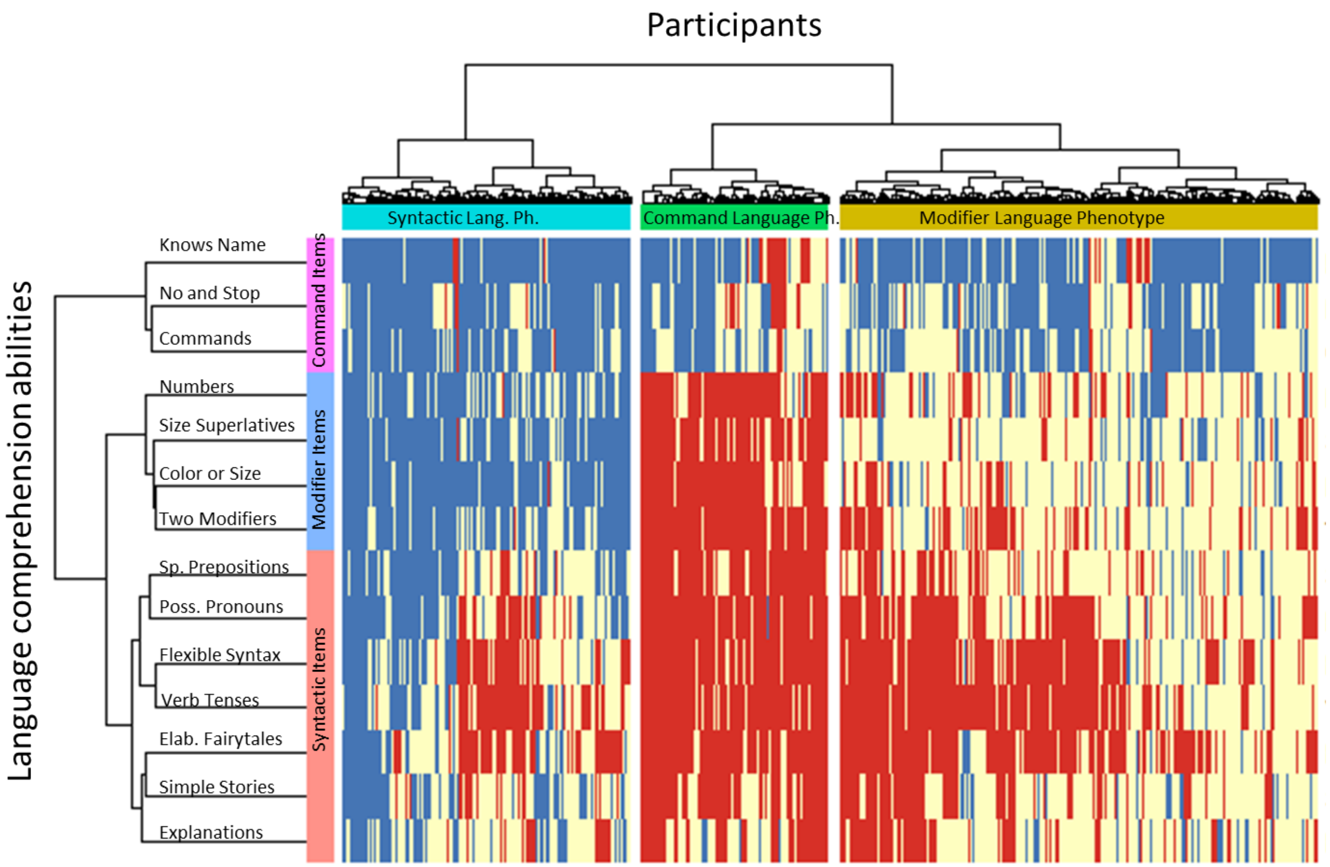

Supplementary Figure 9. Two dimensional heatmap relating 1,604 participants (**of 12 to 21 years of age**) to their language comprehension abilities. The 14 language comprehension abilities are shown as rows. The dendrogram representing language comprehension abilities is shown on the left. Participants are shown as 1,604 columns. The dendrogram representing participants is shown on the top. Blue color indicates the presence of a skill (the "very true" answer), red indicates the lack of skill (the "not true" answer), and white indicates the "somewhat true" answer.

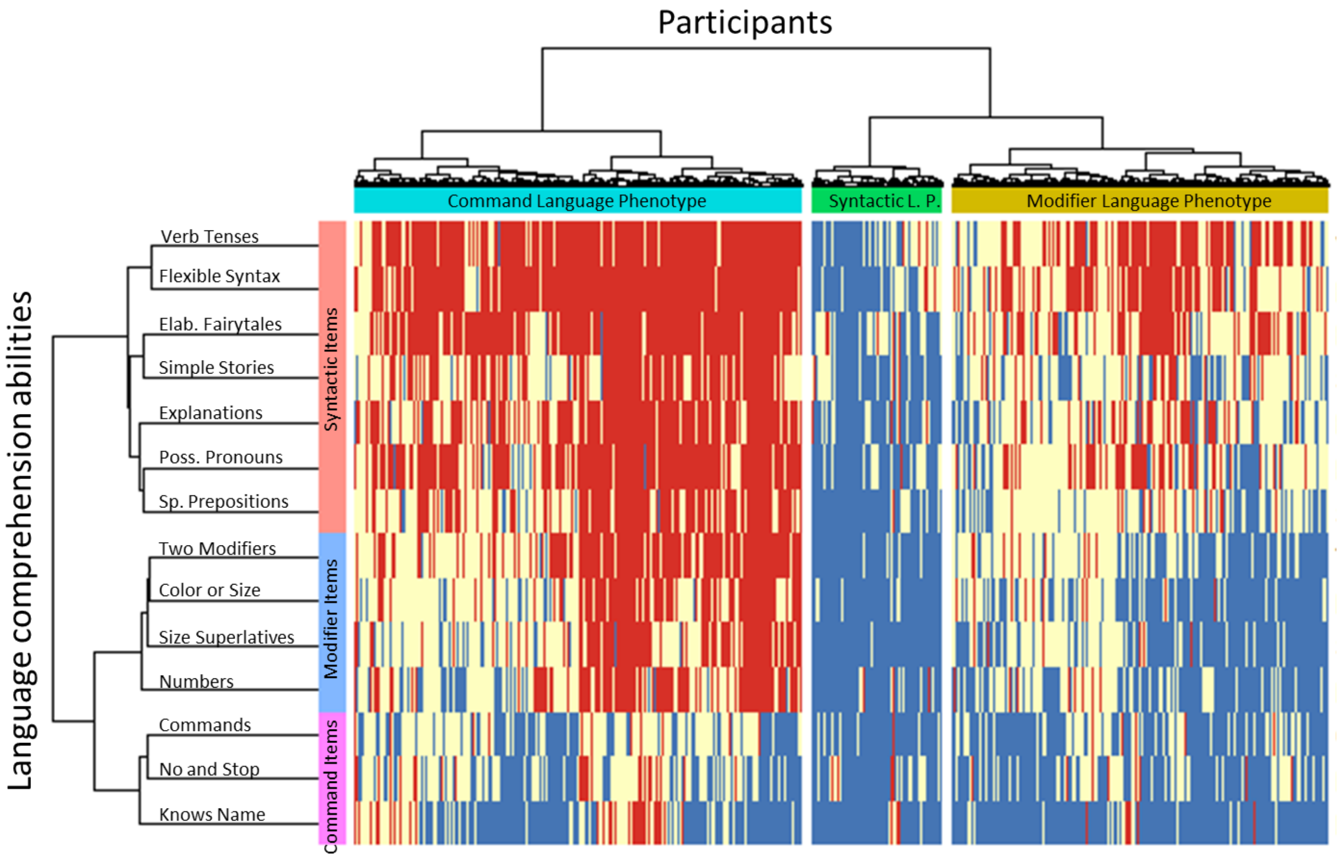

Supplementary Figure 10. Two dimensional heatmap relating 31,845 participants (of 4 to 21 years of age, **first evaluation**) to their language comprehension abilities. The 14 language comprehension abilities are shown as rows. The dendrogram representing language comprehension abilities is shown on the left. Participants are shown as 31,845 columns. The dendrogram representing participants is shown on the top. Blue color indicates the presence of a skill (the “very true” answer), red indicates the lack of skill (the “not true” answer), and white indicates the “somewhat true” answer.

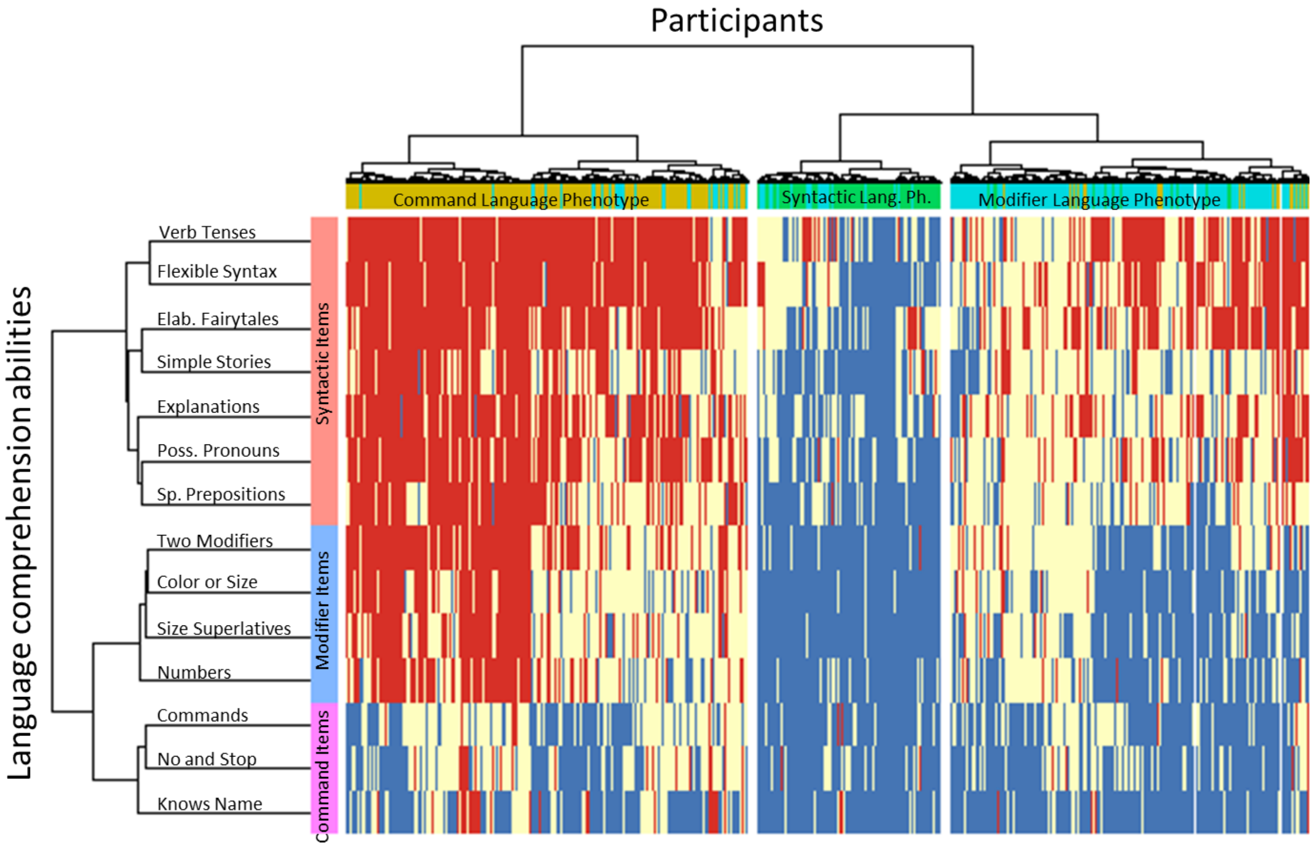

Supplementary Figure 11. Two dimensional heatmap relating 31,845 participants (of 4 to 21 years of age, last evaluation) to their language comprehension abilities. This heatmap uses **Manhattan distance metric** unlike heatmaps above that use Euclidean distance metric. The 14 language comprehension abilities are shown as rows. The dendrogram representing language comprehension abilities is shown on the left. Participants are shown as 31,845 columns. The dendrogram representing participants is shown on the top. Blue color indicates the presence of a skill (the “very true” answer), red indicates the lack of skill (the “not true” answer), and white indicates the “somewhat true” answer.

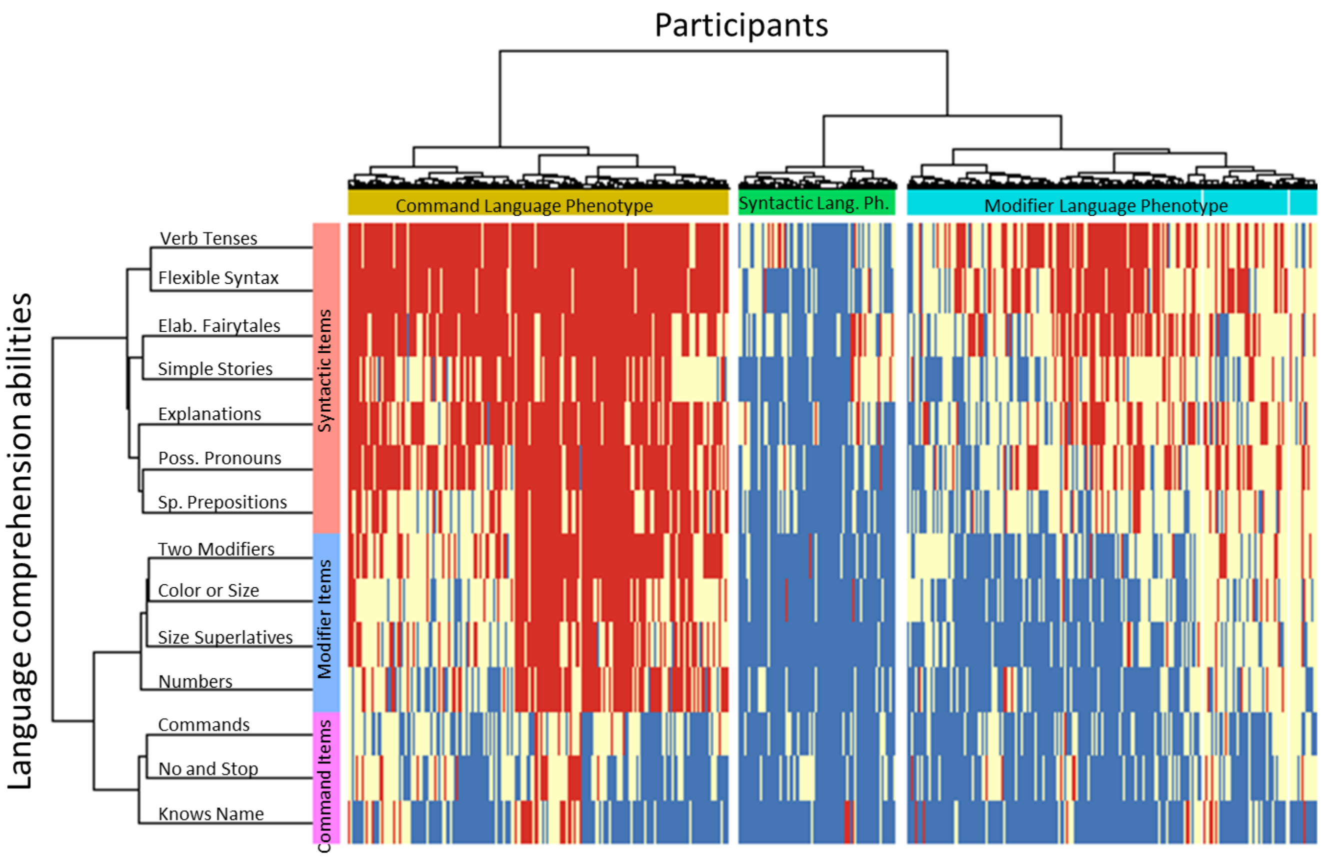

Supplementary Figure 12. Two dimensional heatmap relating 31,845 participants (of 4 to 21 years of age, last evaluation) to their language comprehension abilities. This heatmap uses **Minkowski distance metric**. The 14 language comprehension abilities are shown as rows. The dendrogram representing language comprehension abilities is shown on the left. Participants are shown as 31,845 columns. The dendrogram representing participants is shown on the top. Blue color indicates the presence of a skill (the “very true” answer), red indicates the lack of skill (the “not true” answer), and white indicates the “somewhat true” answer.

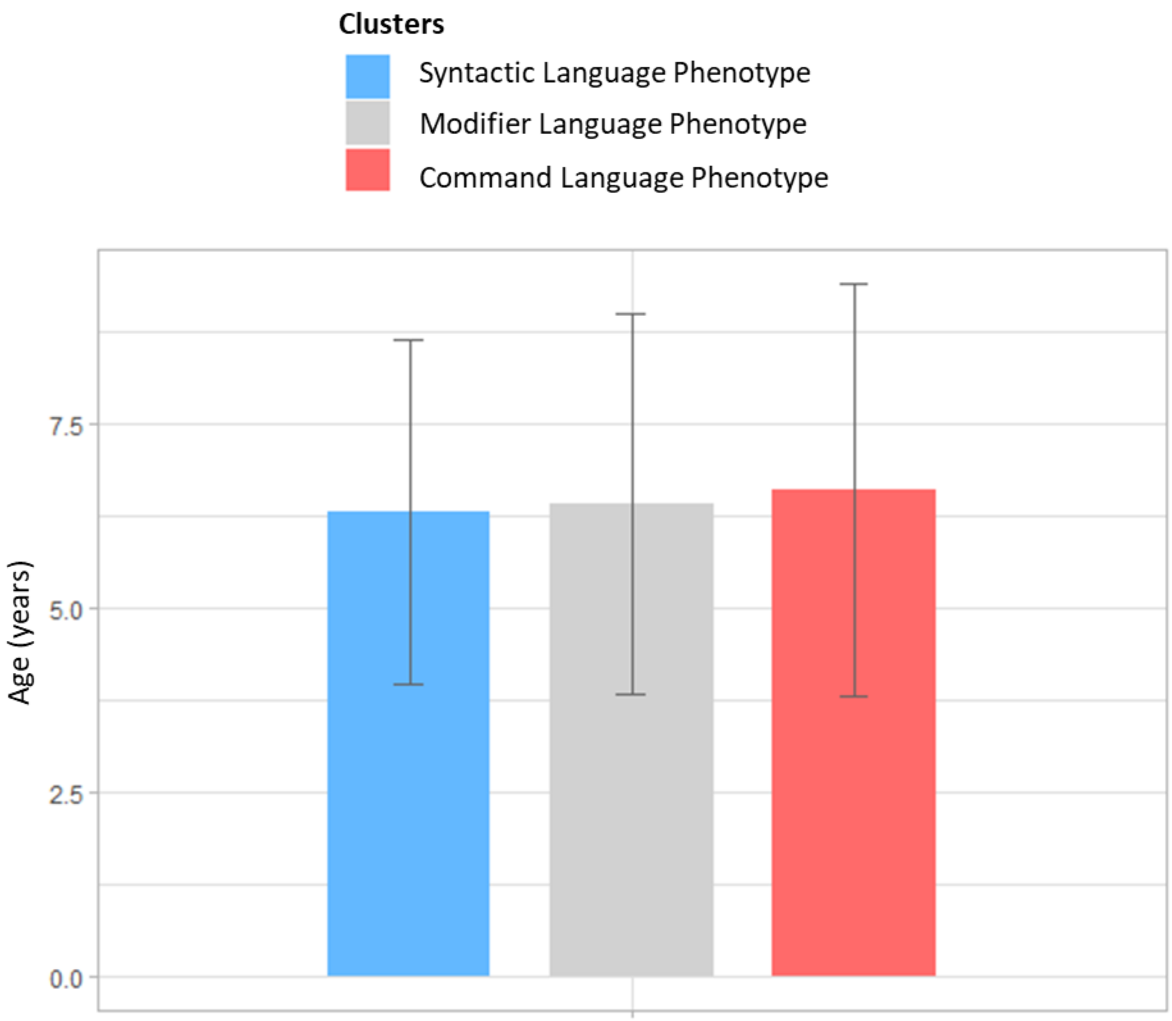

Supplementary Figure 13. Language comprehension phenotype clusters show no statistically significant difference in age. Error bars show standard deviation.

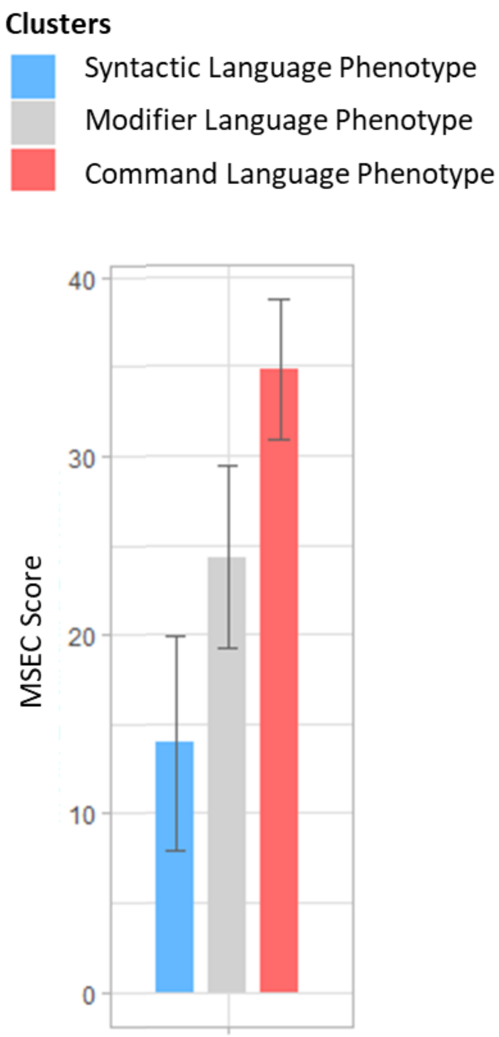

Supplementary Figure 14. Language comprehension phenotype clusters show significant differences in MSEC score (t-test: $p < 0.0001$). Lower scores indicate milder symptoms. Error bars show standard deviation.

Supplementary Table 7: Language comprehension phenotype clusters show significant differences in properties that were not used for clustering, such as expressive language, sociability, sensory awareness, and health ($p < 0.0001$). Lower scores indicate milder symptoms.

Participant Cluster	Expressive Language (Max score=28)	Sociability (Max score=40)	Sensory Awareness (Max score=36)	Health (Max score=75)	MSEC score (Max score=40)
**Syntactic Language Phenotype**	6.3(5.3)	9.4(8.3)	7.2(5.7)	12(8.8)	13.9(6)
**Modifier Language Phenotype**	11.8(5.5)	12.3(7.5)	13.0(6.0)	15.9(9.6)	24.3(5.1)
**Command Language Phenotype**	18.3(5.3)	15.5(7.4)	18.8(6.5)	18.5(10.3)	34.8(3.9)

Supplementary Table 8: Language comprehension phenotype clusters show significant differences in properties that were not used for clustering, such as expressive language, sociability, sensory awareness, and health. Pair-wise comparisons between clusters, t-test, p-value < 0.0001.

	Expressive Language	Sociability	Sensory Awareness	Health	MSEC score
**Syntactic vs. Modifier cluster**	t=-54.584 df=13044	t=-16.546 df=12196	t=-55.859 df=13326	t=-23.742 df=13705	t=-101.000 df=11404
**Modifier vs. Command Cluster**	t=-80.951 df=20773	t=-37.874 df=19765	t=-67.955 df=21470	t=-20.282 df=20863	t=-130.910 df=20692
**Syntactic vs. Command cluster**	t=-127.830 df=12032	t=-48.031 df=10491	t=-118.830 df=12927	t=-43.767 df=13006	t=-204.770 df=10219

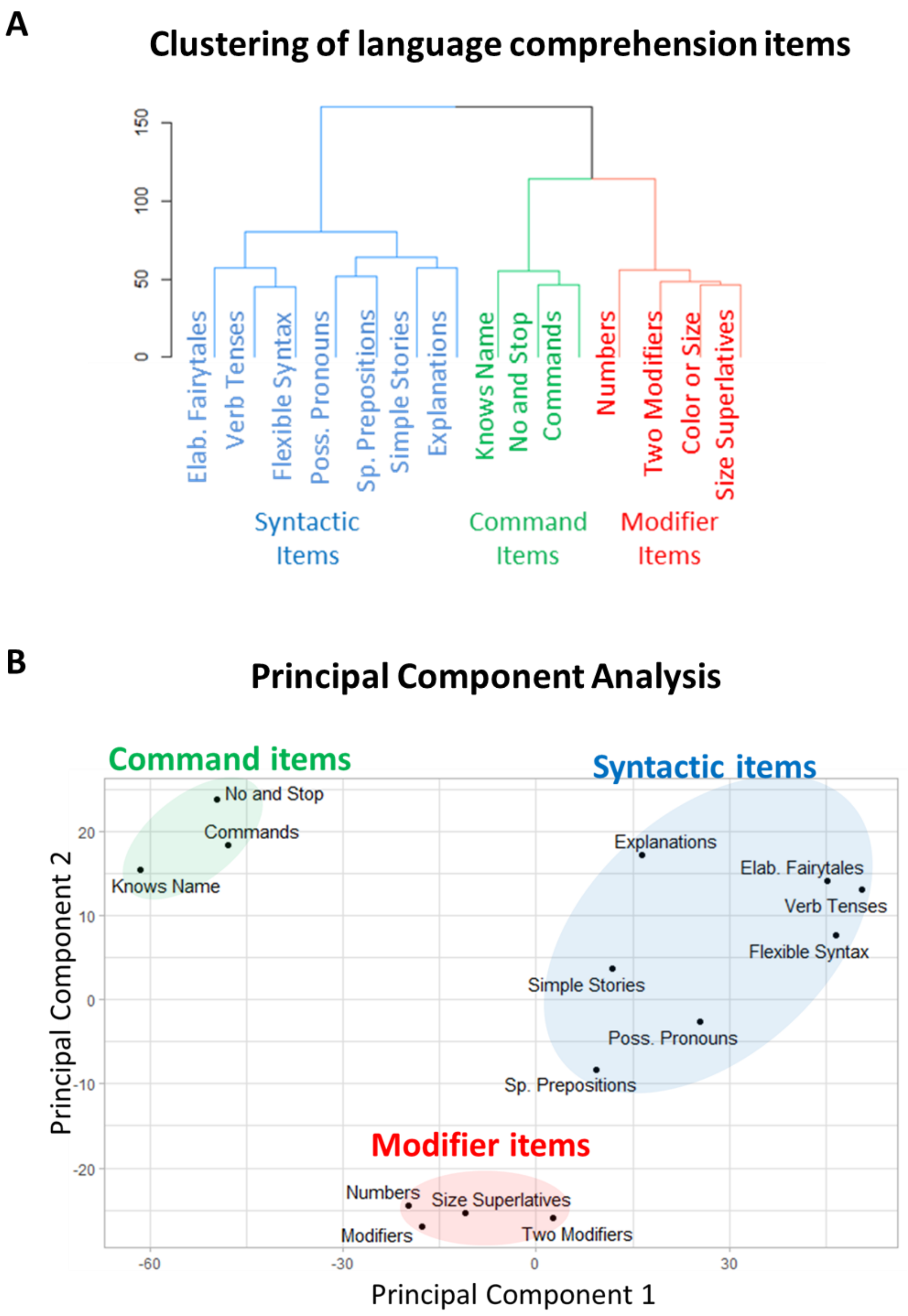

Supplementary Figure 14. Clustering analysis of language comprehension items limited to 6,444, English-speaking participants residing in the USA. (A) The dendrogram representing the unsupervised hierarchical clustering of language comprehension abilities. (B) Principal component analysis of the 14 language comprehension abilities shows clear separation between command, modifier, and prepositional items. Principal component 1 accounts for 47.2% of the variance in the data. Principal component 2 accounts for 12.5% of the variance in the data.

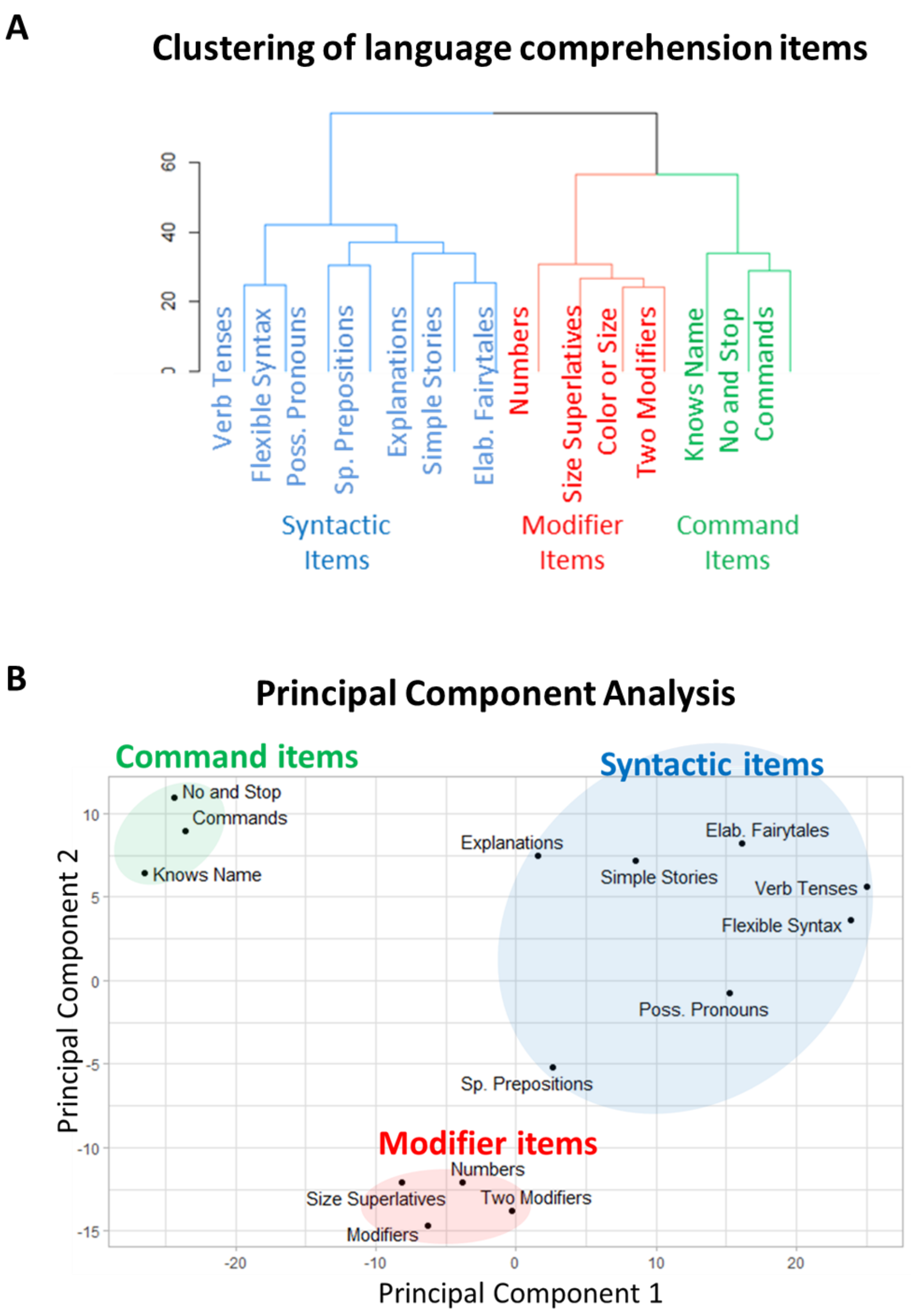

Supplementary Figure 15. Clustering analysis of language comprehension items limited to 6,444, Spanish-speaking participants residing in the USA. (A) The dendrogram representing the unsupervised hierarchical clustering of language comprehension abilities. (B) Principal component analysis of the 14 language comprehension abilities shows clear separation between command, modifier, and prepositional items. Principal component 1 accounts for 38.7% of the variance in the data. Principal component 2 accounts for 12.2% of the variance in the data.

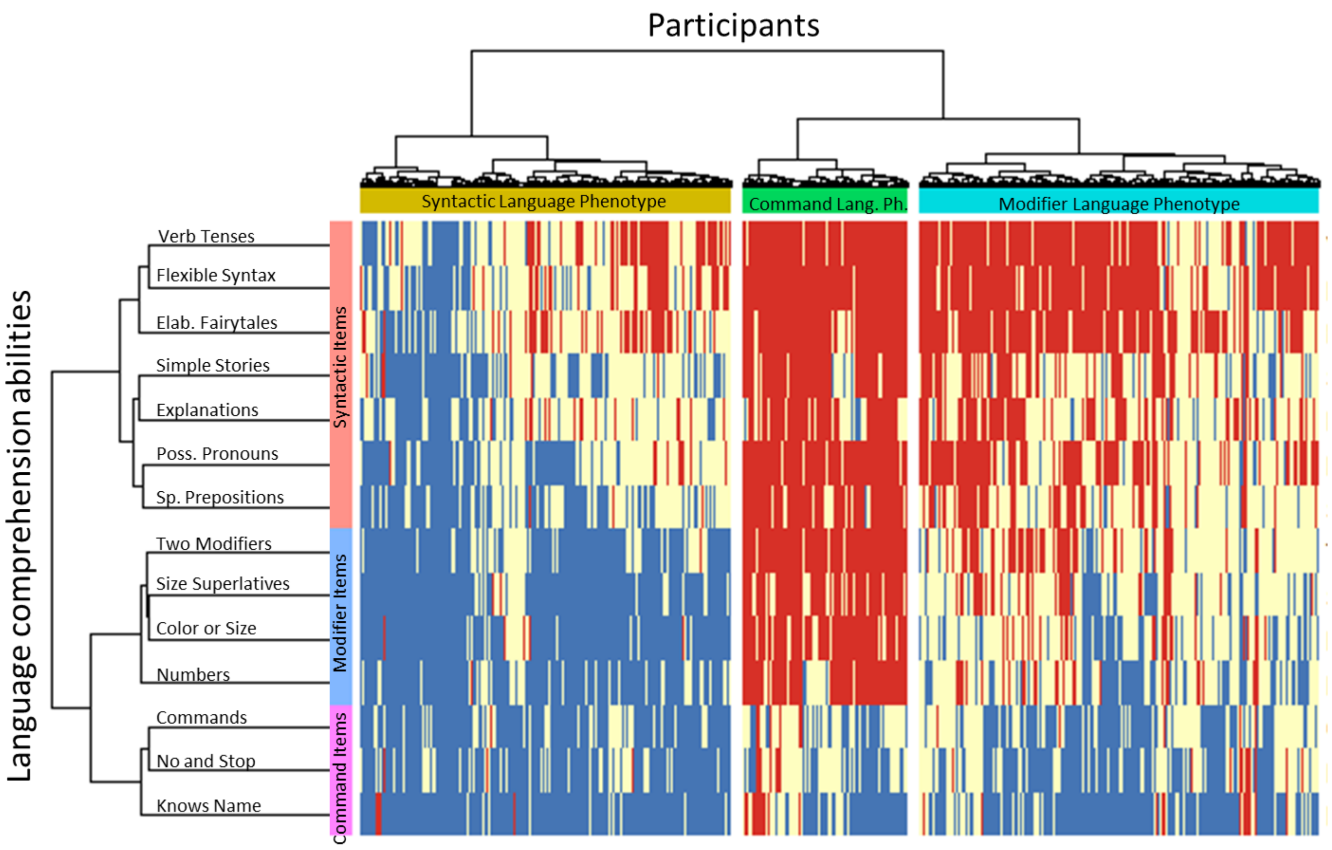

Supplementary Figure 16. Two dimensional heatmap relating 6,444 English-speaking participants residing in the USA to their language comprehension abilities. The 14 language comprehension abilities are shown as rows. The dendrogram representing language comprehension abilities is shown on the left. Participants are shown as 6,444 columns. The dendrogram representing participants is shown on the top. Blue color indicates the presence of a skill (the “very true” answer), red indicates the lack of skill (the “not true” answer), and white indicates the “somewhat true” answer.

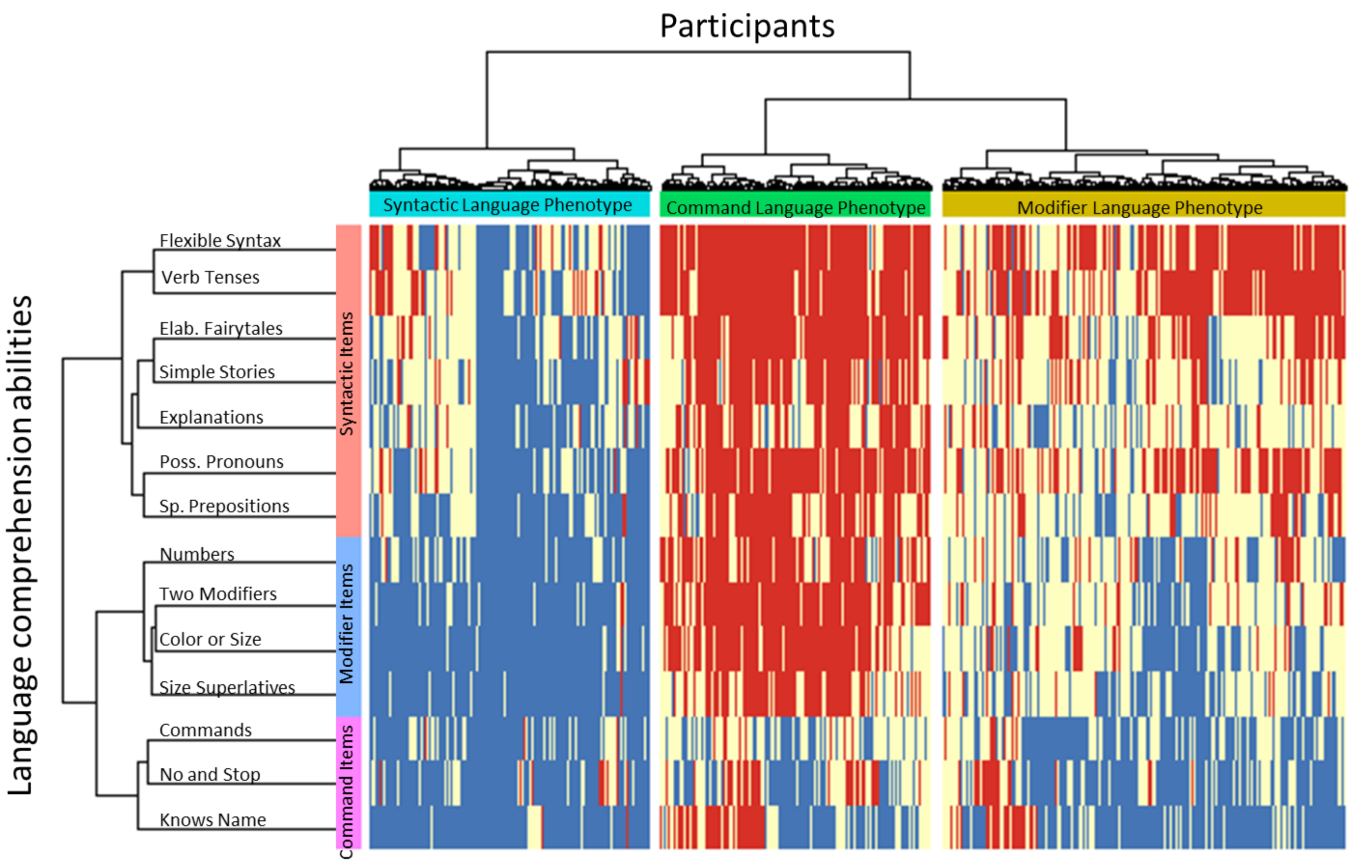

Supplementary Figure 17. Two dimensional heatmap relating 1,771 Spanish-speaking participants residing in the USA to their language comprehension abilities. The 14 language comprehension abilities are shown as rows. The dendrogram representing language comprehension abilities is shown on the left. Participants are shown as 1,771 columns. The dendrogram representing participants is shown on the top. Blue color indicates the presence of a skill (the “very true” answer), red indicates the lack of skill (the “not true” answer), and white indicates the “somewhat true” answer.
